# Palladium(0) NHC complexes: a new avenue to highly efficient phosphorescence†
†Electronic supplementary information (ESI) available. CCDC 1021492 and 1021493. For ESI and crystallographic data in CIF or other electronic format see DOI: 10.1039/c4sc03914a



**DOI:** 10.1039/c4sc03914a

**Published:** 2015-04-02

**Authors:** Adam F. Henwood, Mathieu Lesieur, Ashu K. Bansal, Vincent Lemaur, David Beljonne, David G. Thompson, Duncan Graham, Alexandra M. Z. Slawin, Ifor D. W. Samuel, Catherine S. J. Cazin, Eli Zysman-Colman

**Affiliations:** a Organic Semiconductor Centre , EaStCHEM School of Chemistry , University of St Andrews , St Andrews , Fife KY16 9ST , UK . Email: eli.zysman-colman@st-andrews.ac.uk ; http://www.zysman-colman.com ; Fax: +44-(0)1334-463808 ; Tel: +44-(0)1334-463826; b EaStCHEM School of Chemistry , University of St Andrews , St Andrews , Fife KY16 9ST , UK . Email: cc111@st-andrews.ac.uk ; Fax: +44-(0)1334-463808 ; Tel: +44-(0)1334-464808; c Organic Semiconductor Centre , SUPA School of Physics and Astronomy , University of St Andrews , North Haugh , St Andrews , Fife KY16 9SS , UK . Email: idws@st-andrews.ac.uk; d Service de Chimie des Matériaux Nouveaux & Centre d'Innovation et de Recherche en Matériaux Polymères , Université de Mons – UMONS/Materia Nova , Place du Parc, 20 , B-7000 MONS , Belgium; e WestCHEM Department of Pure and Applied Chemistry and Centre for Molecular Nanometrology , University of Strathclyde , 295 Cathedral Street , Glasgow , G1 1XL , UK

## Abstract

We report the first examples of highly luminescent di-coordinated Pd(0) complexes. Five complexes of the form [Pd(L)(L′)] were synthesized, where L = IPr, SIPr or IPr* NHC ligands and L′ = PCy_3_, or IPr and SIPr NHC ligands. The photophysical properties of these complexes were determined in degassed toluene solution and in the solid state and contrasted to the poorly luminescent reference complex [Pd(IPr)(PPh_3_)]. Organic light-emitting diodes were successfully fabricated but attained external quantum efficiencies of between 0.3 and 0.7%.

## Abstract 2

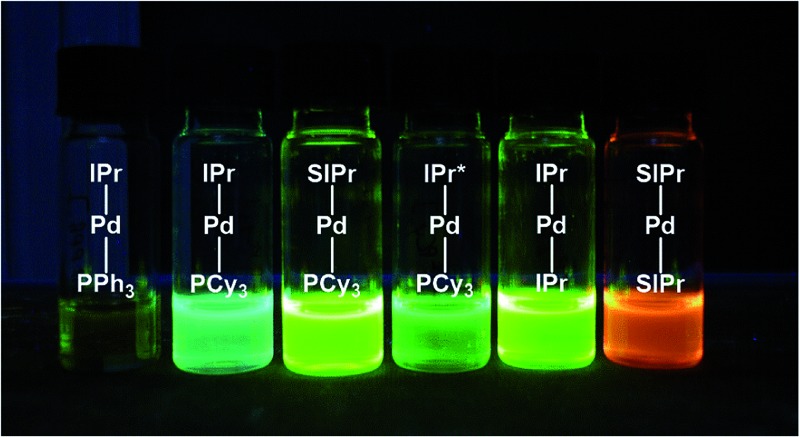
We report the first examples of highly luminescent di-coordinated Pd(0) complexes.

## Introduction

A number of crucial design features need to be considered in order to obtain high performance organic light-emitting diodes (OLEDs). Chief amongst these is the development of bright emitters capable of recruiting 100% of the excitons in an operational device. Phosphorescent molecules satisfy this criterion although emission lifetimes need to be sufficiently short in order to mitigate undesired quenching processes that can reduce the overall efficiency of these types of compounds in the bulk solid.^1^ Color tunability of the material across the visible spectrum is required in order to achieve white light emission, which is a necessary feature for large area lighting applications.^2^ Only a few types of phosphorescent materials have been extensively explored in the context of solid-state lighting that simultaneously satisfy these two key requirements of phosphorescence and color tunability.

By far the most popular and successful class of metal complexes that have been studied so far are based on iridium(iii). The high spin–orbit coupling (SOC) constant of iridium^3^ gives concomitantly short emission lifetimes (*τ*_e_) and high photoluminescence quantum yields (*Φ*_PL_). Facile functionalisation of the ligand scaffold of the prototypical *fac*-Ir(C^N)_3_ complex (where C^N is a monoanionic cyclometalating bis-chelate such as 2-phenylpyridinato) employed for these devices can lead to emission spanning the visible spectrum with *Φ*_PL_ near unity.^4^ However, in spite of these advantages, iridium is incredibly scarce – it is the third rarest transition metal in the Earth's crust^5^ – making the search for alternatives to iridium crucial if large scale implementation of OLED lighting technology is to be realised.

With this scarcity in mind, alternative emitters have been explored in light-emitting devices, including those based on Re(i),^6^ Ru(ii),^7^ and Os(ii).^7c,8^ However, until now these materials have failed to match iridium on device stability,^6^ color tunability^9^ or excited state lifetimes.^7*c*^ So far, only platinum(ii) and copper(i) complexes have proven to be competitive with iridium as phosphorescent materials for solid-state lighting (SSL).

The greater abundance of copper and its significantly lower cost than most other photophysically active metals make it an attractive option, and experimental results both in solution^10^ and in devices^11^ have been encouraging, particularly for green emitters.^12^ Two broad classes of Cu(i) complexes have been explored: multinuclear Cu(i) dimers and clusters;^13^ and more recently, sterically congested mononuclear three- or four-coordinate Cu(i) complexes bearing a combination of N^N, N^P, P^P and *N*-heterocyclic carbene (NHC) ligands.^12,14^ Apart from the obvious benefit of using copper for its low cost, interest is burgeoning in the use of Cu(i) for device applications owing to its capacity to recruit triplet excitons in spite of its small SOC constant (*ξ* = 829 cm^–1^).^14c,15^ This is made possible by the capacity of most (but not all)^14*a*^ Cu(i) complexes to thermally populate the S_1_ state from the T_1_ state in a fashion that allows for thermally activated delayed fluorescence processes to occur (TADF).

However, the stability of mononuclear Cu complexes can be an issue. For example, it is common for these complexes to undergo ligand exchange as a result of their lability. Additionally, tetrahedral four-coordinate complexes can distort to a square planar geometry in the excited state, allowing for nucleophilic attack and the subsequent formation of a “pentacoordinated exciplex” species that undergoes rapid emission quenching and reduces device stability.^11^ Additionally, the lack of bright red copper emitters still remain a major issue.^11^

Besides Cu(i), there has been considerable research into cyclometalated platinum(ii) complexes. These complexes are highly stable, and show comparable microsecond *τ*_e_ values and color tuning properties to Ir(iii). Synthetic versatility in the design of ligand scaffolds for decoration about the platinum center^16^ has sought to take advantage of these properties, leading to reports of highly luminescent complexes bearing multidentate ligands,^17^ NHC-cyclometalating ligands,^18^ azolato-based pseudo-cyclometalating ligands,^19^ among others,^20^ for OLED applications. However, square planar Pt(ii) compounds have the propensity to aggregate through metallophilic interactions that induce undesirable deactivation pathways or excimer formation that reduce color purity.^7*c*^

**Fig. 1:**
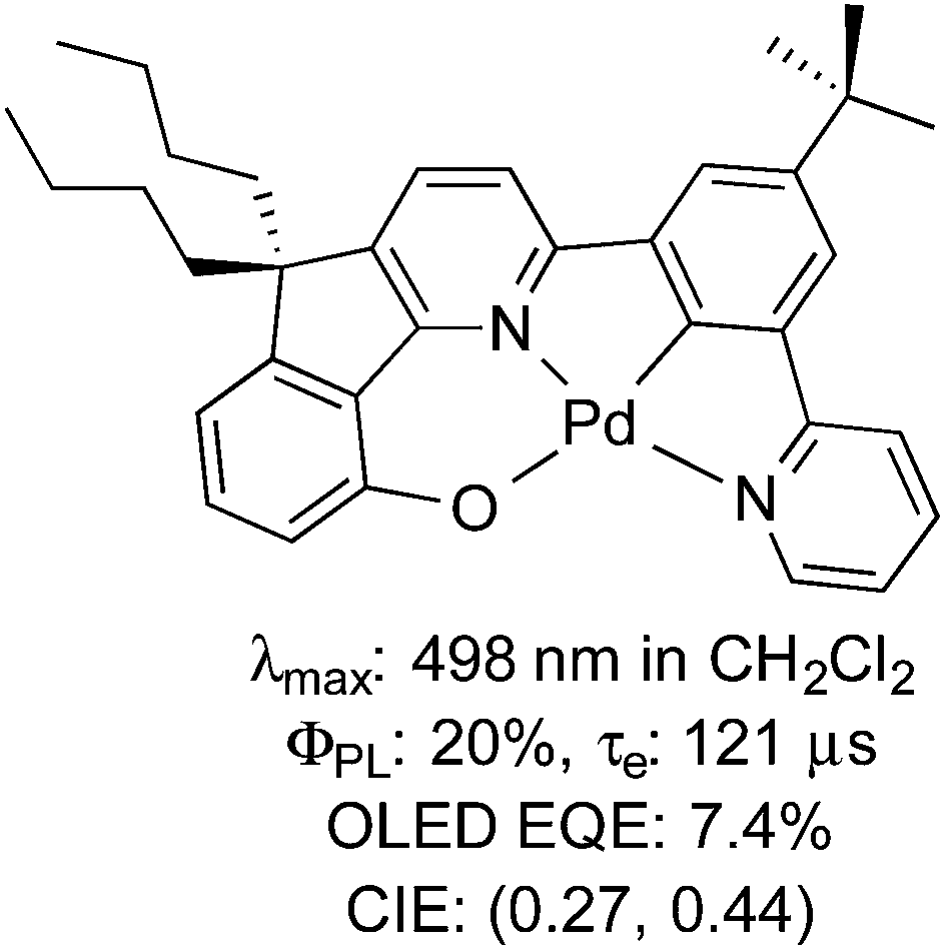
Example of a Pd(ii) complex incorporated into an OLED.^21*d*^

Despite its analogous coordination chemistry with platinum, one metal severely underrepresented in terms of its photophysical properties is palladium. In the case of Pd(ii), this is likely attributable to the much smaller ligand field splitting for Pd(ii) than that of Pt(ii), which results in facile population of the d_*x*^2^–*y*^2^_ antibonding orbital and thus efficient deactivation of the excited state by twisting into a tetrahedral-type *D*_2d_ excited state geometry.^21^ Accordingly, studies into the luminescence of Pd(ii) are sparse and there is only one example of which we are aware where a Pd(ii) complex has been adopted as the emitter in an OLED using vacuum deposition techniques (Fig. 1), although even in this case, the *τ*_e_ of this complex (121 μs) is likely to be too long for widespread commercialisation of this material for OLED applications.^21*d*^

**Fig. 2:**
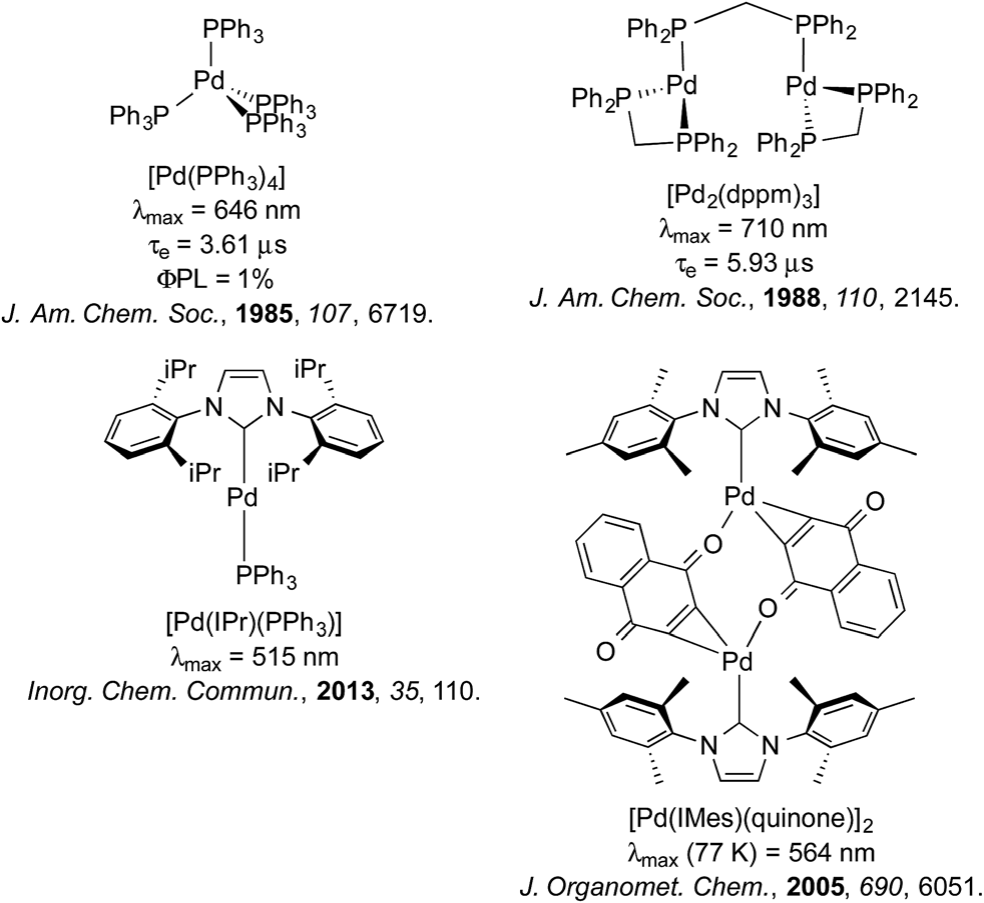
Selection of luminescent Pd(0) complexes bearing phosphines and/or NHCs previously reported.

On the other hand, Pd(0) complexes, while receiving vast attention for their impressive catalytic properties,^22^ have been explored even more sparingly than Pd(ii) in a photoluminescent context. This is perhaps surprising, since Pd(0), bearing a d^10^ electronic structure, has no accessible d–d metal centered (MC) states^23^ that might rapidly deactivate the excited state in a manner similar to Pd(ii). However, the instability of Pd(0) compounds with respect to oxygen^24^ has perhaps dissuaded exploration of the photoluminescence properties of these compounds. Work has largely focussed on luminescence studies of Pd(0) phosphine complexes such as [Pd(PPh_3_)_3_],^24^ [Pd(PPh_3_)_4_],^25^ and [Pd_2_(dppm)_3_] (dppm = 1,1-bis(diphenylphosphino)methane), which turn out to be poorly emissive.^25^ Tsubomura and co-workers have reported a series of tetracoordinated Pd(0) disphosphine complexes that emitted strongly (12 ≤ *Φ*_PL_ ≤ 38%) in the IR region.^26^ More recently, the same group reported a series of poorly luminescent di- and tricoordinated Pd(0) phosphine complexes with emission maxima spanning from blue to orange (*λ*_max_ = 485–700 nm).^27^ Replacement of one of the phosphine ligands with a bulky IPr *N*-heterocyclic carbene (NHC) in [Pd(IPr)(PAr_3_)] also resulted in very weakly emissive complexes (IPr = *N*,*N*′-bis(2,6-(diisopropyl)phenyl)imidazol-2-ylidene).^28^ A dimeric palladium complex bridged by two quinone-type ligands and capped by an NHC was found to be non-emissive at room temperature and photochemically unstable.^29^ Consequently, no Pd(0) complexes as of yet have been reported to be operational in a lighting device. Fig. 2 summarises the photophysics of a selection of these complexes.

Unsurprisingly, a large number of luminescent Pd(0) complexes reported contain NHC ligands, given their suitability as ligand motifs for luminescent applications has been demonstrated extensively across a variety of other metals. Their exceptionally strong σ-donating capabilities, where applicable, serve to push deleterious metal-centered (^3^MC) states to very high energies, making radiative decay pathways more favourable and thus resulting in brightly emissive complexes, while their reasonable π-accepting properties also facilitate metal-to-ligand charge transfer (MLCT) type transitions that are characteristic of many luminescent complexes.^30^ The myriad examples of NHC complexes of other metals includes Cu(i),^31^ Ag(i),^31*a*^ Re(i),^32^ Ir(iii),^33^ Pt(ii),^34^ and Au(i).^35^

**Fig. 3:**
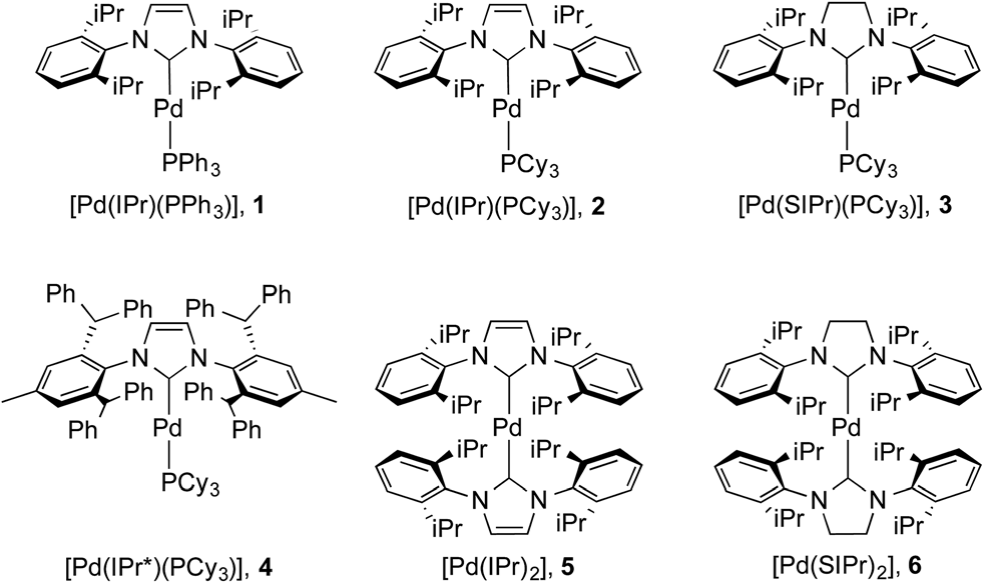
Complexes under investigation in this study.

The issue we explore in this contribution is whether efficient photoluminescence can be obtained from Pd(0) complexes. The present report builds on our previous work on Pd(0) NHC complexes for catalysis^22e,23b,36^ and our recent observations^23*b*^ that many of these complexes (Fig. 3) showed unprecedented intense emission in toluene solution; exceptionally, preliminary absorption and emission data for weakly luminescent [Pd(IPr)(PAr_3_)], **1**, has been reported although not quantified (Ar = Ph, *o*-Tol).^28^ Herein, we report a detailed solution state photophysical study of the first examples of *strongly* emissive Pd(0) complexes, **2–6** (Fig. 3), and compare these to the reference complex **1**. This study showcases the exciting potential of palladium – being more than *fifteen times* more abundant than iridium^37^ – as a potential new class of OLED materials that could substitute for Ir complexes.

## Results and discussion

### Complex synthesis and characterisation

**Fig. 4:**
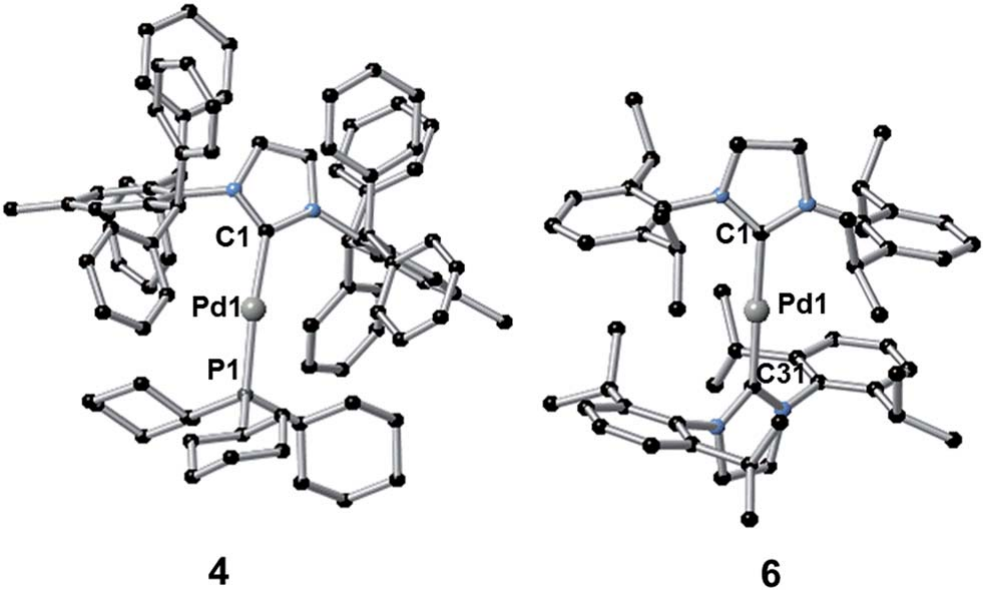
Crystal structures of **4** (left) and **6** (right). Hydrogen atoms have been omitted for clarity. Selected bond lengths [Å] and angles [°]: for **4** Pd1–C1 2.027(4), Pd1–P1 2.2380(11), C1–Pd1–P1 170.91(10); for **6** Pd1–C1 2.030(6) and Pd1–C31 2.015(6), C1–Pd1–C31 178.1(3).

The synthesis of complexes **1–5** was carried out according to the method reported by Nolan.^36*a*^ Reaction of [Pd(η^3^-allyl)(Cl)(NHC)] (allyl = C_3_H_5_) with PR_3_ or NHC in the presence of KO^*t*^Bu gave the desired complexes in 81–85% yield. Complex **6** was synthesized in 66% yield as described in the literature^38^ by reaction of [Pd(μ-Cl)(η^3^-crotyl)]_2_ (crotyl = 3-MeC_3_H_5_) in THF with two equivalents of free SIPr (SIPr = *N*,*N*′-bis[2,6-(diisopropyl)phenyl]imidazolin-2-ylidene). The syntheses of complexes **1–3** and **5–6** have been previously reported, while that of **4** is novel. Complexes **1–3** and **5–6** were characterized by ^1^H and, where applicable, ^31^P{^1^H} NMR, while **4** was characterized by ^1^H, ^13^C{^1^H} and ^31^P{^1^H} NMR and elemental analysis. Suitable crystals for single crystal X-ray diffraction studies were obtained for complexes **4** and **6** after slow diffusion (toluene/isopropanol) at –35 °C (Fig. 4).^39^ Both structures slightly deviate from a linear geometry with NHC–Pd–L bond angle of 170.91(10)° and 178.1(3)° for complexes **4** and **6** respectively. These data are in accordance with the structures reported for related complexes (**1**: 169.49(2)°; **2**: 170.88(2)°; **5**: 175.98(14)°).^40^ The NHC–Pd and NHC–PR_3_ bond lengths for complexes **4** (2.027(4) Å and 2.2380(11) Å, respectively) and **6** (2.030(6) Å) are similar to those reported in the literature for congeners (**1**: 2.0547(8) Å and 2.2100(2) Å, respectively; **2**: 2.0292(9) Å and 2.2212(3) Å, respectively; **5**: 2.026(4) Å). In order to better understand the spatial occupation about the palladium center, percent buried volumes (%*V*_Bur_)^41^ were determined (Fig. S3–S4, S7–S8, S14–S15 and S17†). The range of buried volumes lies between 37.8–48.0% for the NHC and between 30.7–33.3% for the phosphine ligand. There is no correlation between the solution state photophysical properties of these complexes and this structural parameter, however a strong correlation does exist with respect to thin film photophysical properties (*vide infra*).

### UV-vis absorption

**Fig. 5:**
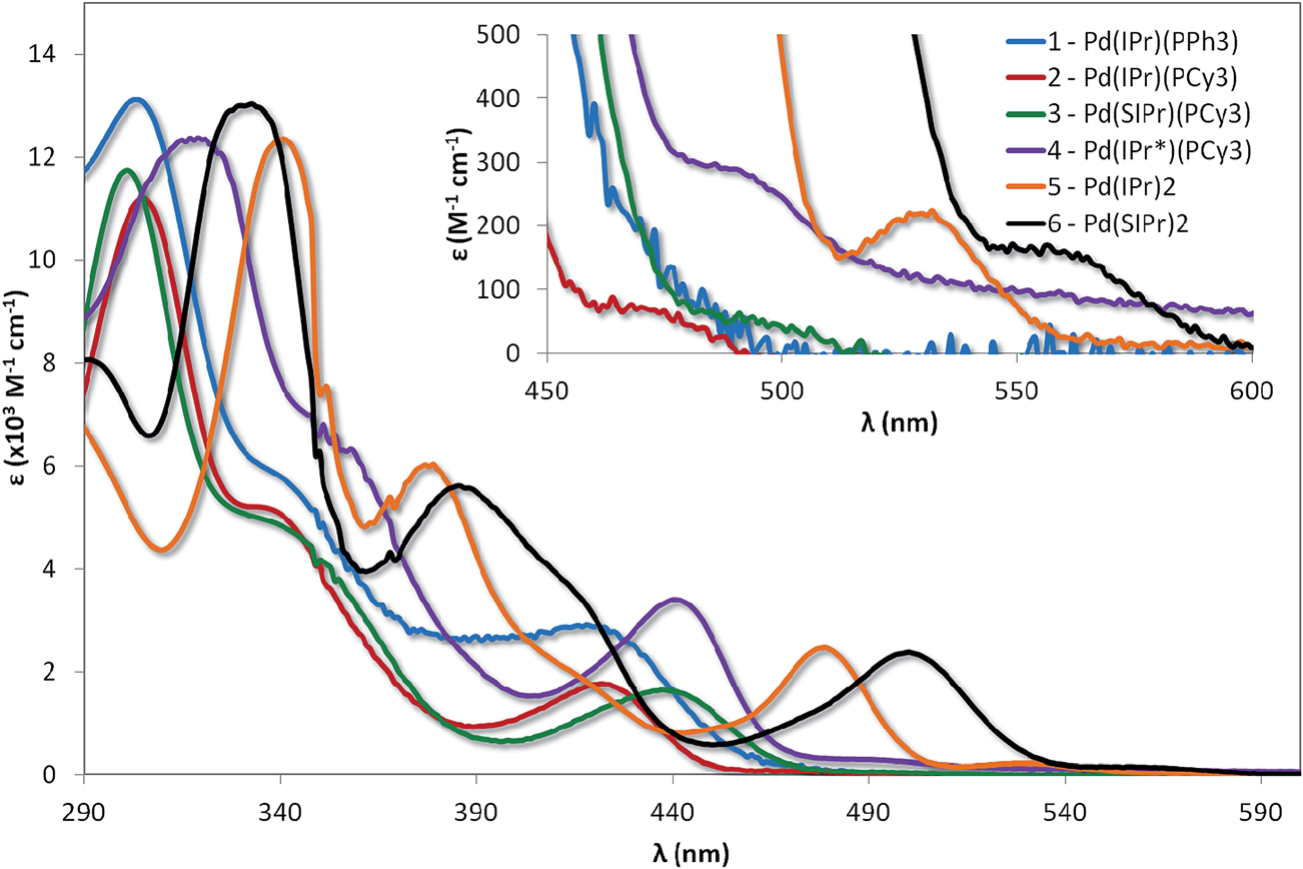
Absorption spectra of complexes explored in this study. Measurements were carried out at 298 K in degassed toluene. Inset shows low energy absorption spectra illustrating the weakly absorbing bands ascribed as direct population of the T_1_ state.

The absorption spectra for the six complexes are shown in Fig. 5 and the molar absorptivity data summarised in Table 1. These complexes are highly reactive towards molecular oxygen, rapidly forming Pd(ii) peroxo adducts upon exposure to O_2_.^22e,23b^ In order to rigorously avoid oxygen exposure, all samples were prepared in the glovebox, with dry degassed toluene used as the solvent of choice to mitigate any solvent coordination of the vacant palladium sites, which has been shown to be possible for linear [Pd(NHC)(PR_3_)] complexes dissolved in solvents such as dichloromethane.^28^

**Table 1:** Absorption data for complexes **1–6** at 298 K

Complex	Absorbance^*a*^ 298 K (nm) [*ε* (×10^3^ M^–1^ cm^–1^)]
**1**	303 [13.1], 344 [5.5], 421 [2.9], 465 [0.24]
**2**	305 [11.1], 338 [5.1], 422 [1.8], 471 [0.07]
**3**	301 [11.7], 342 [4.8], 437 [1.6], 491 [0.06]
**4**	318 [12.3], 353 [6.4], 440 [3.4], 491 [0.29]
**5**	340 [12.3], 378 [6.0], 414sh, 478 [2.5], 529 [0.22]
**6**	332 [13.0], 386 [5.6], 416sh, 500 [2.4], 562 [0.15]

^*a*^All measurements were carried out in anhydrous toluene from samples prepared in a glovebox.

The assignment of the individual absorption bands of each spectrum is made more difficult by the complex nature of the electronic structure of d^10^ complexes, for which any observed MC states are symmetry-allowed. The high-energy bands are usually assigned as typical ^1^π–π* transitions, but the nature of the lower energy bands is less obvious, especially when compared with literature complexes. For example, Tsubomura assigns the lowest energy band for the bent^42^ complex [Pd(PCy)_2_], found at 350 nm, as an MC d_*z*^2^_–p_*z*_ transition.^27^ Similarly, a number of low energy excited states observed for the [Pd_2_(dppm)_3_] dimer (trigonal planar about the palladium centre)^25^ are assigned as a variety of MLCT states: ^1^d*σ–p_σ_ (440 nm), ^3^d_δ_–p_σ_ (500 nm) and ^3^d*σ–p_σ_ (574 nm).^24^ The presence of delocalised NHC ligands with low-lying π-states is likely to result in pronounced MLCT-type transitions taking place for these complexes as well.

Comparing the spectra of **1** with **2** demonstrates that changing the phosphine from PPh_3_ to PCy_3_ does not modulate the electronics in any appreciable fashion, in spite of the expected increased σ-donating capability of the aliphatic phosphine in comparison with the aromatic phosphine. The principal absorption features of **1** (303, 344 and 421 nm) virtually overlap with that of **2** (305, 338, and 422 nm). However, two distinct differences are worth noting: firstly, the magnitude of the extinction coefficients for each of the three bands for **1** are greater than for **2**, suggesting that the increased conjugation of the PPh_3_ ligand contributes to the overall absorptivity of **1**. Secondly, there are weakly absorbing bands at low energy, ascribed as direct population of the triplet state, which are observable for **1** (465 nm) and **2** (471 nm). This band is well resolved in the case of **2**, but much more poorly defined for **1**.

Despite the insensitivity of the electronics of the absorption spectra to phosphine, it is likely that the phosphine ligand contributes to the process, especially when observing the drastic change in the form of the spectra upon replacement of the phosphine for a second NHC. For example, while the molar absorptivity of the band at 340 nm for **5** falls within the regime of the corresponding bands for **1** and **2**, there is a large bathochromic shift of *ca.* 3600 cm^–1^ (35 nm) compared to the corresponding band for **1** (303 nm) and **2** (305 nm). Similar red-shifting of the lower energy absorption bands is also observed for **5**, with the bands at 378 nm and 478 nm red-shifted by ∼3100 cm^–1^ (∼40 nm) and 2800 cm^–1^ (∼55 nm), respectively. In addition, the form of the bands at 344 and 338 nm for complexes **1** and **2** resemble shoulders, rather than the full band at 378 nm observed for complex **5**, suggesting a change in the nature of the transition. Similar changes in the absorption profile of **6** are present when compared to **3**: the band at 332 nm in **6** is moderately more absorptive, consistent with an increased π-network, while there are large red-shifts in three low energy bands in **6** of ∼3100 (31 nm), 3300 (34 nm) and 2883 cm^–1^ (63 nm) compared to those, respectively, found in **3**. Finally, complexes **5** and **6** possess shoulders at 414 and 416 nm, respectively, that are absent in the other complexes, suggesting a new CT transition in these two complexes.

By comparison to the limited change in the absorption spectra observed from **1** to **2**, modifying the NHC leads to small but distinct changes in their spectral profile. For example, the highest energy band for complex **3**, found at 301 nm, is blue-shifted compared to **2** by a modest 436 cm^–1^ (4 nm) upon saturation of the imidazolium NHC backbone, due to reduced delocalisation across the π-network. However, a red-shift of 814 cm^–1^ (15 nm) is observed for the lowest energy band at 437 nm. The red-shift of this ^1^MLCT band is probably due to a lower lying LUMO in **3** compared to **2** as a result of the change in the nature of the NHC ligand from IPr to SIPr. When the steric demand of the NHC ligand is increased significantly in **4**, the absorption spectrum is red-shifted relative to **2** and **3**; the energy differences between the three bands of **2** and of **4** all fall within similar regimes: ∼1000 to 1300 cm^–1^ (7 to 18 nm).

Similar changes in spectral profile are observed from **5** to **6** as were found with **2** to **3**. The magnitude of the energy differences is also quite similar for the two pairs of complexes. For example, the peak at 340 nm for complex **5** is blue shifted by 709 cm^–1^ (8 nm) upon saturation of both NHC backbones (compared with 436 cm^–1^, 4 nm, between **2** and **3**). Similarly, the lower energy band (478 nm for complex **5**) is red-shifted by 921 cm^–1^ (22 nm), which is only a small increase from the 813 cm^–1^ (15 nm) difference between the corresponding bands in **2** and **3**. No noticeable change in molar absorptivity is observed across the spectra when comparing **2** to **3** or **5** to **6** as might be expected for such similar structures.

In order to assist the interpretation of the experimental absorption spectra, time-dependent density functional theory, (TD)-DFT, calculations were performed to examine the nature (spatial localization) and energetics of the frontier molecular orbitals as well as the nature of the low-lying singlet and triplet excited states. Unless otherwise specified, all electronic structure calculations have been performed at the optimized geometry of the ground state.

**Fig. 6:**
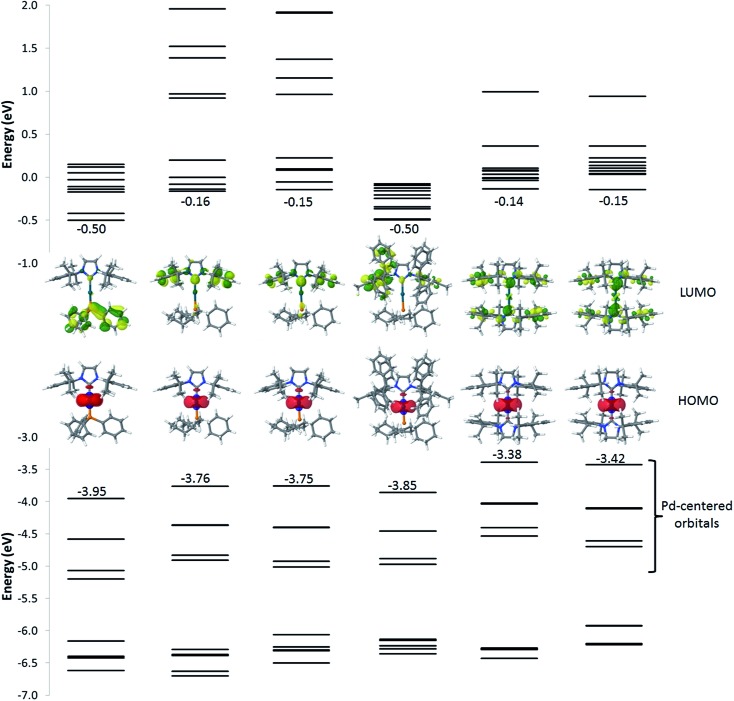
Energy of the frontier electronic levels ranging from HOMO – 9 to LUMO + 9 for all complexes. The pictures correspond to the localization of the HOMO (bottom) and LUMO (top) orbitals.

Fig. 6 and Table S1† summarize the energy and localization of the ten highest occupied and ten lowest unoccupied molecular orbitals (MOs). Regardless of the nature of the ligands, the five highest-energy occupied MOs are palladium-centered. Their energies can however be modulated by the nature of the ligands. For example, replacing PPh_3_ by PCy_3_ (from **1** to **2**, **3**, or **4**) leads to a ∼0.2 eV destabilization of the metal-centred orbitals as a result of the increased σ-donating character of the aliphatic phosphine. For complexes with two NHC ligands (**5** and **6**), the energies of the HOMOs are further destabilized. As for the lowest unoccupied energy levels, they are similar for complexes **2**, **3**, **5** and **6** and localized over the whole NHC ligand for the LUMO yet only on the peripheral aryl rings for LUMO + 1, LUMO + 2 and LUMO + 3. Interestingly, complex **4** exhibits LUMO levels that are likewise localized on the NHC ligand but at lower energy compared to complexes **2**, **3**, **5** and **6** because of an increased delocalization across the π-network present in the IPr* ligand. The nature of the first unoccupied orbitals differs significantly in **1** compared to all other complexes, since the former involve PPh_3_.

**Fig. 7:**
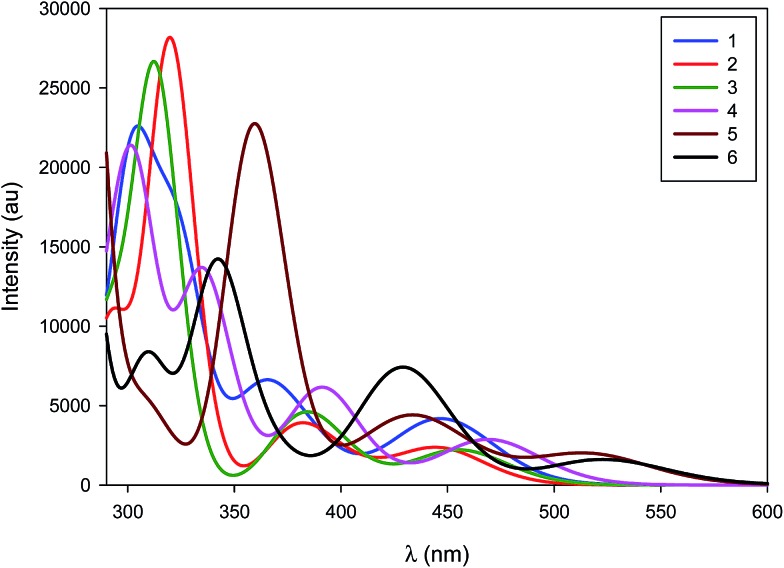
TD-DFT simulated absorption spectra of **1–6**.

The simulated absorption spectra of **1–6** are displayed in Fig. 7. The TD-DFT calculations (i) indicate that all electronic excitations wavelengths longer than 250 nm involve excited states with dominant MLCT character (from the occupied Pd d-centred orbitals to the unoccupied ligand-centred orbitals) (Fig. 6 and Table S1†) and (ii) support the view that the weakly absorbing bands at low energy are due to direct excitation into triplet states (see below). For **1**, all the absorption bands represented in Fig. 6 correspond mainly to the promotion of one electron from the Pd centre to the PPh_3_ group, though there is a minor contribution of the peripheral aryl moieties of the NHC ligand to the band simulated at 305 nm and a very weak contribution for the band at 366 nm. In line with the measured results, the simulated absorption spectra of **2** looks similar to **1** but the nature of the bands is totally different, as expected from the change in the nature of the frontier molecular orbitals. The first two bands at low energy (444 nm and 382 nm) only involve the NHC ligand while PCy_3_ and NHC contribute to the band at 320 nm. For complex **3**, the measured red-shifts of the lowest two optical absorption bands (+15 nm and +4 nm) and the corresponding blue-shift of the third band (–4 nm) as compared to complex **2** are well-reproduced by the calculations (with predicted shifts of +11 nm, +2 nm, and –7 nm, respectively). The nature of the main optical transitions in **3** and **2** is similar, except for the band at 384 nm for which the contribution of the imidazolium ring is drastically reduced in **3**. Saturating the imidazolium ring has therefore only a limited impact on the optical properties of the complexes. Incorporating phenyl rings in IPr* in **4** leads to a systematic red-shift of all the absorption bands compared to **2**; the nature of each of the transitions is however preserved. The red-shifts originate from the stabilization of the first unoccupied levels stemming from an increased π-delocalization. With two NHC ligands on the Pd (complexes **5** and **6**), there is a large red-shift of the absorption bands, which originates from the destabilization of the Pd-centred occupied orbitals. In both compounds, the lowest energy band features a large contribution over the imidazolium unit while the higher energy bands involve the entirety of the NHC ligands (except for the second band of **6** for which the imidazolium segment does not contribute significantly). These results are consistent with those obtained when saturating the imidazolium ring of complex **2**. Overall, the predicted singlet electronic transitions reproduce the measured optical absorption spectra remarkably well, except at low energy where we hypothesize that direct absorption into triplet states occurs. To check this hypothesis, we have calculated the vertical S_0_ → T_1_ transition energy for each of the complexes; these match well the weak low-energy spin-forbidden absorption bands (calculated [measured] maxima at 499 nm [465 nm], 500 nm [471 nm], 516 nm [491 nm], 522 nm [491 nm], 580 nm [529 nm] and 594 nm [562 nm] for **1**, **2**, **3**, **4**, **5** and **6**, respectively). It is interesting to point out that the lowest triplet excited state is in all cases MLCT-like, thus reflecting the nature of the corresponding singlet states.

### Solution state photophysical behavior

**Fig. 8:**
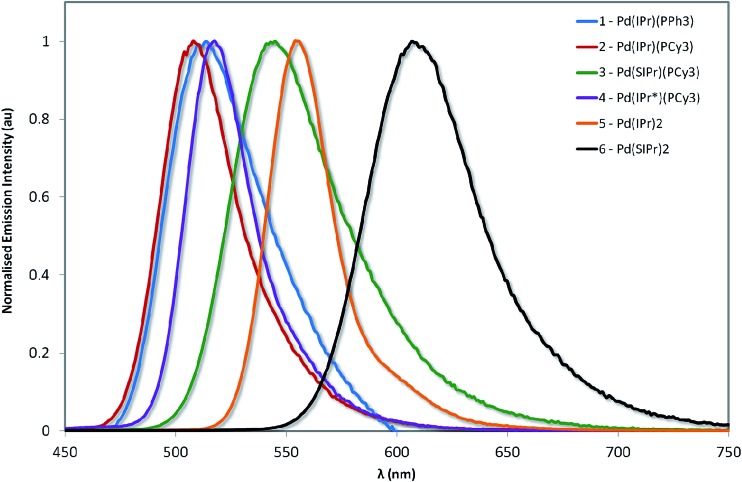
Normalized emission spectra of complexes explored in this study. Measurements were carried out at 298 K in degassed toluene and result from excitation at 360 nm.

The normalized emission spectra of the six complexes are shown in Fig. 8, while the relevant photophysical data are summarised in Table 2. All measurements were carried out on samples prepared in the glovebox using anhydrous toluene.

**Table 2:** Relevant photophysical data for **1–6** in toluene solution

Complex	*λ*_em_ ^*a*^ (nm)	*Φ*_PL_ ^*b*^ (%)	*τ*_e_ ^*c*^ (μs)	*k*_r_ (×10^5^ s^–1^)	*k*_nr_ (×10^5^ s^–1^)
**1**	512	1.0	0.014	7.14	707
**2**	508	16.4	1.56	1.05	5.36
**3**	543	69.0	5.46	1.26	0.57
**4**	515	13.9	0.36 (15%), 1.59 (85%)	—	—
**5**	555	70.1	2.93	2.39	1.02
**6**	608	69.8	5.62	1.24	0.54

^*a*^Measured at 298 K in degassed toluene and excited at 360 nm.

^*b*^Using quinine sulfate as the standard (*Φ*_PL_ = 54.6% in 0.5 M H_2_SO_4_ at 298 K).^43^

^*c*^Excited at 379 nm.

Emission across the family of complexes ranges from 508 to 608 nm. In spite of the relatively small Stokes shifts observed for these complexes, emission is ascribed to phosphorescence on the basis of the microsecond lifetimes observed for all the complexes except **1** and the presence of the heavy palladium center.

On the basis of the unstructured emission profiles, ligand centred (LC) emission, which is common for many other d^10^ complexes, can be ruled out.^44^ The profiles of the emission spectra are broad and unstructured and are thus assigned to metal-to-ligand charge transfer (MLCT) states, similar to other Pd(0) systems and in line with the TDDFT calculations.^24^

The emission energies are virtually unchanged with respect to the nature of the phosphine ligand, with **1** emitting at 512 nm and **2** at 508 nm. This strongly suggests that the phosphine is not significantly involved in the emission. The susceptibility of the electronics to changes in the NHC is much more pronounced. For instance, there is a significant energy difference (1268 cm^–1^, 35 nm) between the emission maxima of **2** and **3**. The increased electronic density conferred by the SIPr NHC serves to red-shift emission, suggesting that it is raising the energy of the metal-localised frontier orbital and reducing the HOMO–LUMO gap. By comparison, the adoption of the bulky IPr* ligand in **4** confers only a modest 268 cm^–1^ (7 nm) red-shift in the emission compared with **2**.

Substitution of the phosphine for a second NHC ligand results in a dramatic red-shift of the emission. The emission of **5** is red-shifted by 1667 cm^–1^ (47 nm) with respect to **2** – illustrative of the increased σ-donation ability of the second NHC ligand compared to phosphine. Interestingly, this observed red-shift is of similar magnitude to that identified between **2** and **3**, suggesting that any π-back-bonding effected by the IPr imidazolium ring is mitigating the overall σ-donating properties of the NHC.

Like the structure-property differences between **2** and **5**, the emission energy of **6** is red-shifted compared to **3**. However, this red-shift is larger (1968 cm^–1^, 65 nm) than the IPr couple, suggesting a cumulative effect from the increased σ-donation of the second NHC and the decreased π-back-bonding of SIPr compared to IPr. Analogously, **6** is red-shifted compared to **5** by 1571 cm^–1^ (53 nm).

Interestingly, the experimentally observed emission wavelengths match extremely well with the theoretical results calculated on the basis of the lowest triplet state optimized geometries (Table 3). The red-shifts upon saturation of the imidazolium ring and for complexes with two NHC ligands are well-reproduced. Quantum-chemical calculations also confirm that the emission process has strong MLCT character involving the NHC ligands, except for **1** for which PPh_3_ is also contributing (Fig. S32†).

**Table 3:** TD-DFT calculated (column 2) and experimental (column 3) emission wavelengths together with the relative deviation with respect to the calculated values (column 4)

Complex	*λ*_em,th_ (nm)	*λ*_em,exp_ (nm)	Deviation (%)
**1**	549	512	7
**2**	553	508	9
**3**	600	543	10
**4**	561	515	9
**5**	619	555	12
**6**	679	608	12

The most important result is that the photoluminescence quantum yields (*Φ*_PL_) of **2–6** are very high, ranging from 14–70%. To the best of our knowledge, **3**, **5** and **6** display the highest quantum yields for any palladium complex to date, be they Pd(0) or Pd(ii). Previously, the highest reported *Φ*_PL_ for a Pd(0) complex is 8% for the complex [Pd_2_(dpam)_3_] (dpam = bis(diphenylarsino)methane), while the brightest Pd(ii) complexes rely on the use of tetradentate ligands such as porphyrins (*Φ*_PL_ = 47%)^45^ or O^N^C^N cyclometalating ligands (*Φ*_PL_ = 22%)^21*d*^ whereby the rigidity imparted by these ligands restricts the capacity for these complexes to undergo distortion in the excited state, facilitating radiative decay as a result.

These high *Φ*_PL_ values can be rationalized from the excited states kinetics. For example, the parent complex **1** is barely emissive, with an overall *Φ*_PL_ value of just 1.0%, and has a notably short excited state lifetime (*τ*_e_) of just 14 ns. The deleteriously large contribution from *k*_nr_ (two orders of magnitude larger than *k*_r_) leads to drastically shortened *τ*_e_ values and thus inefficient emission. By comparison, **2** exhibits a *k*_nr_ that is two orders of magnitude smaller *versus***1**: *τ*_e_ increases to 1.56 μs and *Φ*_PL_ increases to 16.4% in **2**.

Despite very little difference in emission energy between **1** and **2**, there is a marked increase in *Φ*_PL_ observed for **2** over **1**. It is possible that the bulkier PCy_3_ ligand may more effectively conformationally lock the orientation of the NHC to minimize eclipsing interactions, effectively rigidifying the system such that non-radiative decay becomes less efficient.

Complex **3** (*Φ*_PL_ = 69.0%) emits even more efficiently than **2**, which is explained by the order of magnitude reduction in *k*_nr_ for **3** over **2**. Accordingly, the value for *τ*_e_ is larger (5.46 μs). Interestingly, in spite of the added steric bulk present in **4**, this complex is less emissive than both **2** and **3**, with *Φ*_PL_ = 13.9%.

Finally, the bis-NHC complexes **5** and **6** are also very efficient emitters. Counter-intuitively to what might be expected from the energy gap law, virtually no change in *Φ*_PL_ is observed for complex **6** (*Φ*_PL_ = 69.8%) in comparison to **5** (*Φ*_PL_ = 70.0%), in spite of a red-shift in emission of 1571 cm^–1^.

### Solid state photophysical behavior

**Fig. 9:**
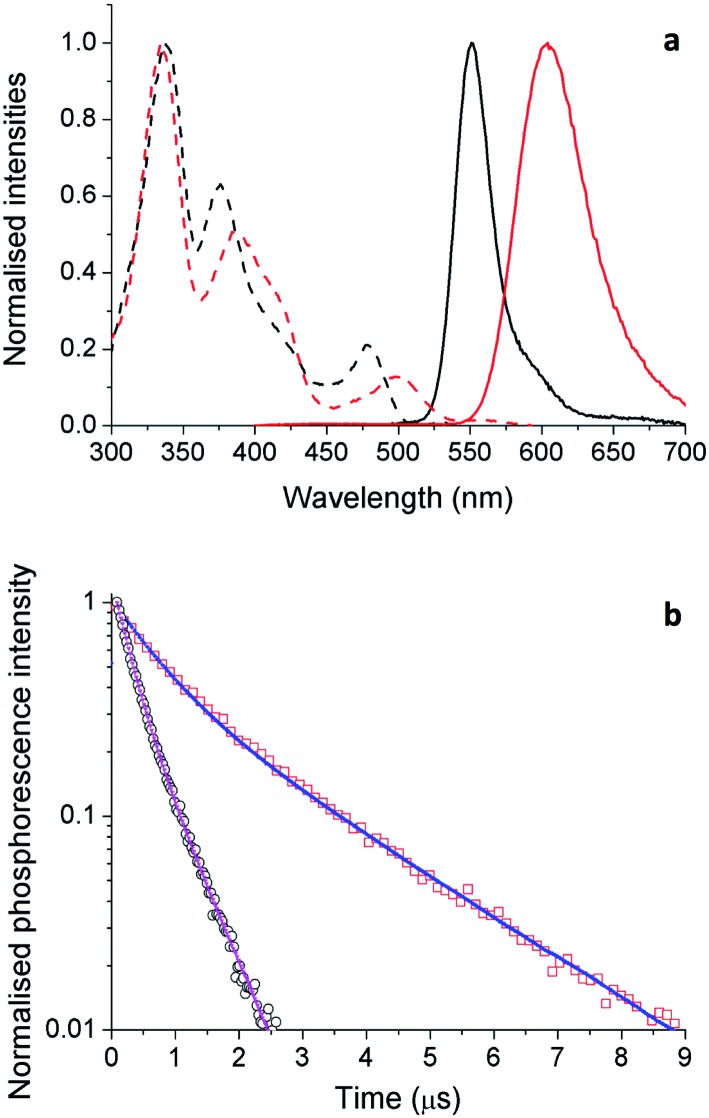
(a) Normalized excitation (dashed) and emission (solid) spectra of **5** (black) and **6** (red) in thin films. (b) Time-resolved PL intensity of materials **5** (blue) and **6** (red) in thin films. The samples were excited at 379 nm.

Although solution measurements give valuable insight into the photophysical properties of these materials it is the thin film properties that are pertinent for identifying lead candidates for electroluminescent devices. We therefore investigated thin film PL behavior next. The thin film excitation and PL spectra are shown in Fig. 9a for complexes **5** and **6**. The thin film spectra for these two complexes are similar to their respective toluene solution spectra. However, for **5**, the solid state emission spectrum is broader than that in solution with a low energy tail evident, which is attributed to aggregate or excimer emission arising from inter-chromophore interactions.

To probe the photophysical properties more closely and the role of inter-chromophore interactions, we measured the *Φ*_PL_ and PL lifetimes in the solid-state (Table 4) for complexes **3**, **5** and **6**, which showed the highest *Φ*_PL_ in solution (*cf.*Table 2). We noticed an important difference in their properties in moving from the solution to the solid state. For **3**, the neat film is almost nonluminescent with a *Φ*_PL_ of only 1.3%. Despite the bulkiness of the ligands, the low *Φ*_PL_ is due to strong inter-chromophore interactions in the film. Substitution of the phosphine ligand for a second NHC ligand partially mitigates this undesired non-radiative pathway. For **5** there is a decrease in *Φ*_PL_ in moving from solution (70%) to the film (10%); however, it is a less pronounced decrease than that observed with **3**. Finally, the film *Φ*_PL_ of **6** was found to be 20.1%. The significant thin film *Φ*_PL_ values correlate with the high %*V*_Bur_ of the NHC ligand (**5**: 42.6%; **6**: 37.8%) indicating the importance of an extremely congested ligand sphere in order to produce bright emitters in the solid state for this family of complexes.

**Table 4:** Relevant photophysical data for **5** and **6** in thin films^*a*^

Complex	*λ*_em_ (nm)	*Φ*_PL_ (%)	*τ*_1_ (μs)	*A*_1_	*τ*_2_ (μs)	*A*_2_
**5**	550	10.1	0.269	0.40	0.645	0.60
**6**	604	20.3	0.698	0.23	2.28	0.77

^*a*^Measured at RT in encapsulated films and excited at 379 nm.

Similar trends are seen in the time-resolved PL (TRPL) measurements. As the light-emitting chromophores are very similar for **5** and **6**, differences in emission lifetime are likely to arise from differences in non-radiative decay rate; *i.e.*, faster decay implies more quenching of PL as a result of inter-chromophore interactions. The TRPL results for **5** and **6** in thin films are shown in Fig. 9b and the fitted parameters are collected in Table 4. The PL decay of **5** is significantly faster than that of **6**. This is consistent with the observed thin film *Φ*_PL_ of these materials, showing strong quenching in the solid state.

### Cyclic voltammetry

**Fig. 10:**
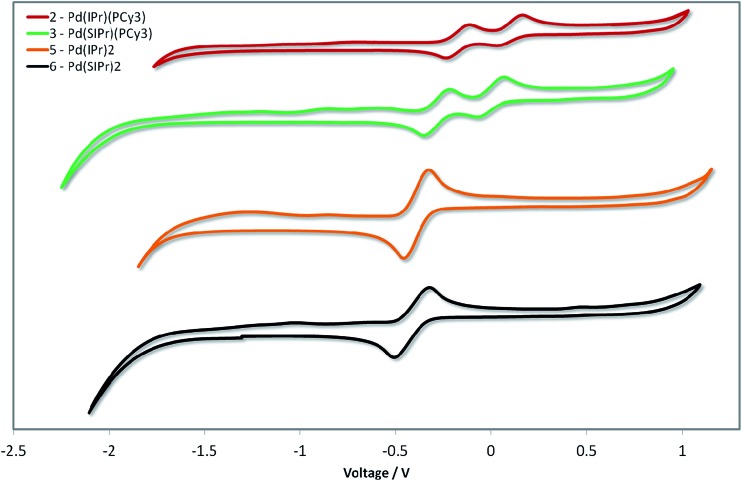
Stacked cyclic voltammograms of **2**, **3**, **5** and **6** in degassed THF using a platinum disk working electrode, platinum wire counter-electrode, a Ag/Ag^+^ pseudo-reference electrode and referenced *vs.* SCE using Fc/Fc^+^ as an internal standard (0.56 V in THF)].^46^ Scan rate: 50 mV s^–1^.

Given the unprecedented photophysical properties demonstrated by these Pd(0) complexes, their electrochemistry was studied by cyclic voltammetry (CV) in order to discern their suitability as materials for OLEDs. Measurements were carried out on the four most promising candidates (**2**, **3**, **5** and **6**) and the relevant data are given in Fig. 10 and in Table 5.

**Table 5:** Relevant electrochemical data for complexes **2**, **3**, **5** and **6**^*a*^

Compound	*E* ox 1/2 (V) [Δ*E*_p_ (mV)]	*E* ox 1/2 (V) [Δ*E*_p_ (mV)]	*E*_HOMO_ ^*b*^ (eV)	*E*_LUMO_ ^*c*^ ^*d*^ (eV)
**2**	–0.17 [114]	0.10 [119]	–4.07	–1.39
**3**	–0.29 [119]	0.00 [115]	–3.95	–1.38
**5**	–0.39 [149]		–3.85	–1.46
**6**	–0.45 [236]		–3.79	–1.53

^*a*^All measurements were performed at 50 mV s^–1^ in deaerated THF solution using Fc/Fc^+^ as an internal standard, and are referenced with respect to SCE (Fc/Fc^+^ = 0.56 V in THF).^46^

^*b*^ 
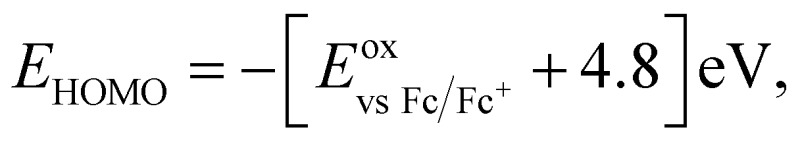
where 
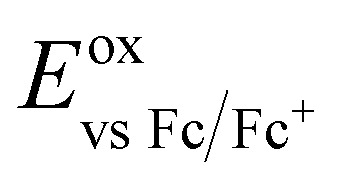
.^47^

^*c*^ *E*_LUMO_ = *E*_HOMO_ + *E*_0,0_ eV.

^*d*^ *E*_0,0_ estimated from the intersection point of the absorption and emission spectra at 298 K in toluene.

The CV traces are divided into two families. The heteroleptic complexes **2** and **3** show two closely spaced waves in the region of 0.1 to –0.4 V. Based on the pronounced reversibility of these waves, they are attributed to sequential one electron oxidations of the palladium centre from Pd(0) to Pd(i) and then Pd(ii), which has been a phenomenon observed in several Pd(L)_2_ systems, where L is a phosphine ligand.^48^ Taking into account the dependency of the low energy absorption bands on the nature of the NHC ligand, the first oxidation wave, and hence the HOMO, is assigned to the Pd^0/I^ redox couple with contribution from the NHC ligand. The second oxidation wave is assigned to the Pd^I/II^ redox couple with contribution from the phosphine ligand based in part on similar oxidation potentials reported for Pd(0)(PR_3_)_*n*_ systems.^48a,49^ Indeed, the higher HOMO energy observed for the more electron rich **3** over **2** corroborates these assignments.

In contrast to the heteroleptic complexes, the homoleptic complexes **5** and **6** show only a single reversible oxidation wave in the region of –0.4 V. Based on the larger peak-to-peak separation, Δ*E*_p_, and greater currents, we tentatively assign these waves as two non-resolved single electron oxidations oxidation waves. In certain cases – particularly when the ligand scaffold about the palladium center is the same – resolving the two single-electron oxidation waves from Pd(0) to Pd(ii) is non-trivial, requiring careful control of thermodynamic and kinetic parameters of the system to distinguish these events.^48*c*^ Once again, the SIPr containing complex, **6**, possesses a higher HOMO level than the IPr analog **5**. The CV data are consistent with the structure–property relationships established by absorption spectroscopy (*vide infra*).

### OLED devices

**Fig. 11:**
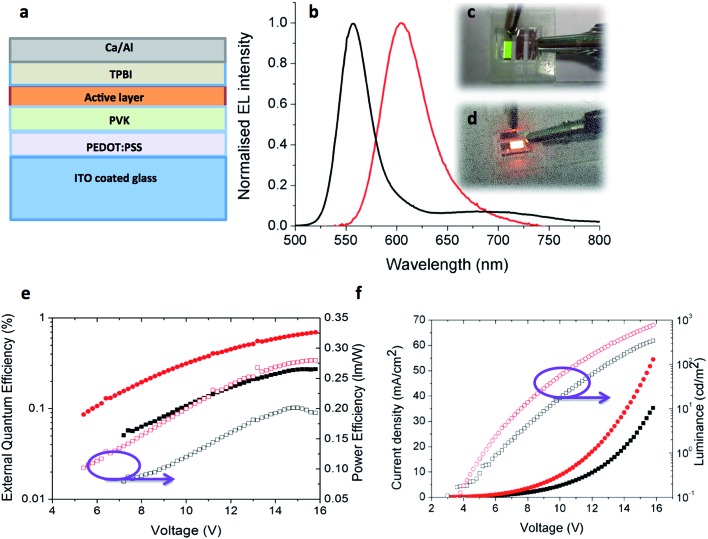
(a) Device architecture for OLEDs using **5** or **6** as the emissive layer. (b) Normalized electroluminescent emission spectra of **5** (black) and **6** (red) in thin films. Photograph of the working OLED with (c) **5** (d) **6**. (e) External quantum efficiency and power efficiency *versus* applied voltage for OLEDs. (f) Current density and luminance *versus* applied voltage for OLEDs made with **5** (black) and **6** (red).

With the most promising candidates identified in terms of their high thin film *Φ*_PL_, OLED devices using **5** and **6** were fabricated and tested. The device architecture is shown in Fig. 11a, having ITO/PEDOT:PSS (40 nm)/PVK (35 nm)/active layer (40 nm)]/TPBI (60 nm)/Ca (20 nm)/Al (100 nm) structure where **5** or **6** act as the active layer. Except for TPBI and the contacts, all the layers were deposited by solution processing methods. Device performance is summarized in Table 6.

**Table 6:** OLED performance data

Complex	Turn on voltage (V)	Current density (mA cm^–2^)	Luminance (cd m^–2^)	EQE (%)	Power efficiency (lm W^–1^)
**5**	8.2	35.2	342	0.28	0.20
**6**	6.2	54.5	766	0.70	0.28

The EL spectra are very similar to the PL spectra of the neat films as shown in Fig. 11b, meaning that in both cases the complexes are emitting from the same excited state. Given that in a device the charge carriers are most likely to recombine on the lowest energy sites, the fact that the EL is not red-shifted relative to the PL is a further indication that the Pd complexes bearing two NHC ligands do not suffer from strong aggregation effects. Fig. 11e shows the external quantum efficiency and power efficiency of the devices. An upper limit on the potential EQE of a device can be estimated from the PLQY of the film and the out-coupling of light (normally taken to be 20% given the refractive index of typical organic materials) giving 2% for **5** and 4% for **6**.^50^ For the OLED using **5** the measured EQE is less than 0.3% in comparison to the OLED using **6** where the EQE reaches to 0.7%, both of which are much lower than materials of their solid state PLQY could achieve. This is likely to be due to an imbalance of charge injection and transport in the device. Fig. 11f shows the current–voltage–luminance characteristics of devices. The turn on voltages for both devices is similar. The key parameters of the devices are highlighted in Table 6. These results suggest the performance of the devices can be optimized further by improving the charge balance.

## Conclusions

In summary, six di-coordinated neutral palladium(0) complexes bearing a combination of NHC and PR_3_ ligands have been synthesized and had their full solution photophysics characterized. In spite of their inherent reactivity, their exceptional photophysics makes them exciting candidates for potential OLED applications. In particular, complexes **3**, **5** and **6**, with *Φ*_PL_ values close to 70% in solution, are the most efficient emitters of any palladium complexes reported to date. This is likely attributable to the lack of d–d MC states that tend to plague the emissive properties of conventional Pd(ii) complexes. Just as significantly, these complexes also satisfy the criteria of color tunability and short lifetimes, with emission spanning from blue-green to orange, and *τ*_e_ values of the most emissive complexes ranging from 1.56 to 5.62 μs. By fulfilling the three main criteria of bright, tunable and short-lived emission, this work has shown that palladium(0) NHC complexes are attractive candidates for OLED applications. OLEDs were successfully fabricated using **5** and **6** and in these initial studies the EQEs were 0.3 and 0.7%, respectively. The EL study shows that further refinement in charge balance is necessary in order to improve the performance of the OLED devices.

## Experimental section

### Photophysical measurements

All samples were prepared in the glovebox using dry toluene with varying concentrations on the order of μM. Samples were sealed within an in-house made Schlenk-cuvette containing a Young tap, and were placed under an argon atmosphere. Absorption spectra were recorded at RT using a Shimadzu UV-1800 double beam spectrophotometer. Molar absorptivity determination was verified by linear least-squares fit of values obtained from at least three independent solutions at varying concentrations with absorbance ranging from 1.26 × 10^–4^ to 3.43 × 10^–5^ M.

As with the samples for absorption, the sample solutions for the emission spectra were prepared in toluene solution in the glovebox and sealed within an in-house made Schlenk-cuvette containing a Young tap, before placing under an argon atmosphere. Steady-state emission, excitation and time-resolved emission spectra were recorded at 298 K using an Edinburgh Instruments F980. All samples for steady state measurements were excited at 360 nm while samples for time-resolved measurements were excited at 378 nm. Photoluminescence quantum yields were determined using the optically dilute method.^51^ Four dilutions were prepared with absorbances of *ca.* 0.1 0.075, 0.05 and 0.025, respectively. The Beer–Lambert law was found to be linear at the concentrations of the solutions. The emission spectra were then measured and for each sample, linearity between absorption and emission intensity was verified through linear regression analysis and additional measurements were acquired until the Pearson regression factor (*R*^2^) for the linear fit of the data set surpassed 0.9. Individual relative quantum yield values were calculated for each solution and the values reported represent the slope value. The equation *Φ*_s_ = *Φ*_r_(*A*_r_/*A*_s_)(*I*_s_/*I*_r_)(*n*_s_/*n*_r_)^2^ was used to calculate the relative quantum yield of each of the sample, where *Φ*_r_ is the absolute quantum yield of the reference, *n* is the refractive index of the solvent, *A* is the absorbance at the excitation wavelength, and *I* is the integrated area under the corrected emission curve. The subscripts s and r refer to the sample and reference, respectively. A solution of quinine sulfate in 0.5 M H_2_SO_4_ (*Φ*_r_ = 54.6%) was used as the external reference.^43^ Solid-state PLQY measurements were measured in an integrating sphere under a nitrogen purge using a Hamamatsu C9920-02 luminescence measurement system.

Time-resolved luminescence measurements were performed using the time-correlated single photon counting technique, with excitation at 375 nm from a pulsed Picoquant GaN laser diode and an instrument response of 250 ps. The PL decay in each case was measured at a wavelength corresponding to the peak of the emission spectrum.

### Quantum-chemical calculations

The singlet and triplet ground-state geometries of the Pd(0) complexes have been optimized at the Density Functional Theory (DFT) level, starting from the X-ray structures when available. The chosen exchange correlation functional is the widely used B3LYP functional^52^ and the basis set for the description of the electrons of the non-metallic atoms is 6-31G**^53^ while the LANL2DZ basis set^54^ is used for the metallic centre.^55^ The PCM (Polarizable Continuum Model)^56^ scheme has been coupled to all (TD)-DFT calculations to account for solvent (toluene) effects. Within this model, the solute is embedded in a shape-adapted cavity surrounded by the solvent implicitly described by a dielectric continuum which characterized by a dielectric constant (*ε* = 2.37 for toluene). The characterization of the nature of the lowest-lying singlet and triplet excited states involved in the absorption spectra relies on time-dependent density functional theory (TD-DFT) performed on the basis of the singlet optimized ground-state geometries and using the same functional and basis set. The emission wavelengths were also calculated at the TD-DFT level but on the basis of the triplet ground-state geometry. All (TD)-DFT calculations were performed within the Gaussian09 package.^57^ The isosurface plots in Fig. S32† were generated by the Jmol program^58^ by combining for each atom the LCAO coefficients in all unoccupied molecular orbitals involved in the TD-DFT description of the excited state and their weights in the wavefunctions.

### Electrochemical measurements

Cyclic voltammetry (CV) measurements were performed on an Electrochemical Analyzer potentiostat model 600D from CH Instruments. Solutions for cyclic voltammetry were prepared in the glovebox using degassed THF. Tetra(*n*-butyl)ammoniumhexafluorophosphate (TBAPF_6_; *ca.* 0.1 M in THF) was used as the supporting electrolyte. An Ag/Ag^+^ electrode (silver wire in a solution of 0.1 M KCl in H_2_O) was used as the pseudo-reference electrode; a Pt electrode was used for the working electrode and a Pt electrode was used as the counter electrode. The redox potentials are reported relative to a saturated calomel electrode (SCE) electrode with a ferrocene/ferrocenium (Fc/Fc^+^) redox couple as an internal reference (0.56 V *vs.* SCE).^46^

### Devices fabrication

The organic light emitting diode was prepared on 12 mm × 12 mm glass substrates with indium tin oxide (ITO) top anode layer of 120 nm. The substrates were carefully cleaned by ultrasound-assisted cleaning in water, acetone and isopropanol for 15 minutes. All the organic layers on top of the anode were prepared by spin coating inside the glovebox where the oxygen and water content was reported less than 0.1 ppm. All the organic materials and solvents were used without further purification. All the solvents used were purchased from Sigma Aldrich. The buffer layer poly(3,4-ethylenedioxythiophene) poly(styrenesulfonate) (PEDOT:PSS) was purchased from Clevios and was used without further purification. Polymer poly (9-vinylcarbazole) (PVK) was purchased from Sigma Aldrich and Organic small molecule 2,2′,2′′-(1,3,5-benzenetriyl)-tris(1-phenyl-1-*H*-benzimidazole) (TPBI) was purchased from Luminescence Technology Corp.

The organic light emitting diode structure was: ITO coated glass, a 40 nm layer of PEDOT:PSS by spin coating at 4000 rpm for 60 seconds and baked on a hot plate at 120 °C for 15 minutes. A hole-transport layer of 35 nm-thick poly(*N*-vinylcarbazole) (PVK) was spin-coated on the PEDOT:PSS layer and baked at 80 °C for 2 h in a nitrogen glovebox. An emissive layer of the Pd complex was spin coated on top of PVK layer by spinning at 2000 rpm. An electron-transport layer of 60 nm-thick TPBI was deposited without a shadow mask in a thermal evaporator. A cathode of Ca/Al (20 nm/100 nm) was then deposited on the TPBI layer in the same vacuum system using a shadow mask. The concentration of polymer PVK in chlorobenzene was 10 mg ml^–1^ and of compound complex **6** was 5 mg ml^–1^ in degassed toluene solvent. OLED consisted of three pixels of dimensions 2 mm × 4 mm. The active area of the OLEDs was encapsulated by microscopic slides using UV curable epoxy (Norland 68) by shining 365 nm from low power UV lamp for 3 minutes.

## Supplementary Material

Supplementary information

Crystal structure data

Crystal structure data
