# Supplementary material for: Palladium(0) NHC complexes: a new avenue to highly efficient phosphorescence
Source: Chem Sci. 2015 Apr 2;6(5):3248–61. doi: 10.1039/c4sc03914a (PMC5657412; doi:10.1039/c4sc03914a)
Supplement: Supplementary file 1 [file SC-006-C4SC03914A-s001.pdf]

Electronic Supplementary Material (ESI) for Chemical Science.
This journal is © The Royal Society of Chemistry 2015

Palladium(0) NHC Complexes: A New Avenue to Highly Efficient Phosphorescence

Adam F. Henwood[a‡], *Mathieu Lesieur*[b‡], *Ashu K. Bansal*[c‡], *Vincent Lemaur*[d], *David Beljonne*[d], *David G. Thompson*[e], *Duncan Graham*[e], *Alexandra M. Z. Slawin*[b], *Ifor D. W. Samuel*[c]*, *Catherine S. J. Cazin*[b]* and *Eli Zysman-Colman*[a]*

[a] Organic Semiconductor Centre, EaStCHEM School of Chemistry, University of St Andrews, St Andrews, Fife, UK, KY16 9ST, Tel: +44-1334 463826; Fax: +44-1334 463808; E-mail: eli.zysman-colman@st-andrews.ac.uk; URL: http://www.zysman-colman.com
[b] EaStCHEM School of Chemistry, University of St Andrews, St Andrews, Fife, UK, KY16 9ST, Tel: +44-1334 464808; Fax: +44-1334 463808; E-mail: cc111@st-andrews.ac.uk;
[c] Organic Semiconductor Centre, SUPA School of Physics and Astronomy, University of St Andrews, North Haugh, St Andrews Fife, KY16 9SS, UK; E-mail: idws@st-andrews.ac.uk
[d] Service de Chimie des Matériaux Nouveaux & Centre d'Innovation et de Recherche en Matériaux Polymères, Université de Mons - UMONS / Materia Nova, Place du Parc, 20, B-7000 MONS, Belgium
[e] WestCHEM Department of Pure and Applied Chemistry and Centre for Molecular Nanometrology, University of Strathclyde, 295 Cathedral Street, Glasgow, G1 1XL, UK

‡ These authors contributed equally to the work.

SUPPORTING INFORMATION

Table of contents:

	Pages
Synthesis, structural characterization and $\%V_{Bur}$ of complexes in this study	S2-S21
Normalised UV-vis, excitation, emission and lifetime spectra of individual complexes in this study	S22-S32
Supplementary computational output	S34

General procedure for the synthesis of complexes [Pd(NHC)(PR_3)] and [Pd(IPr)$_2$], 1-5.[1] In a Schlenk flask, [Pd(η^3-allyl)(Cl)(NHC)] (0.175 mmol) and KO^{t}Bu (0.193 mmol) were dissolved in ^{i}PrOH (3 mL). The reaction mixture was stirred for 2 minutes and NHC' (0.175 mmol) or PR_3 (0.175 mmol) was added portion-wise. The reaction mixture was then stirred for the required reaction time ([Pd(IPr)(PPh_3)] (2 h), [Pd(IPr)(PCy_3)] (4 h), [Pd(SIPr)(PCy_3)] (5 h), [Pd(IPr*)(PCy_3)] (5 h), [Pd(IPr)$_2$] (4 days)).

Workup:

For [Pd(IPr)(PPh_3)], [Pd(IPr*)(PCy_3)] and [Pd(IPr)$_2$], the precipitate was collected by filtration, dissolved in benzene and any insoluble material was removed by filtration. The supernatant solution was dried under vacuum to give the desired micro-analytically pure compound.

For [Pd(IPr)(PCy_3)] and [Pd(SIPr)(PCy_3)], the solution was filtered to remove any insoluble material and the supernatant was dried under vacuum to give the desired micro-analytically pure compound.

[Pd(IPr)(PPh_3)], 1: yellow powder. **Yield**: 85%. **^{1}H NMR (300 MHz, C_6D_6, 298K):** δ (ppm) = 7.52- 7.44 (m, 6H, $C_{Ar}H$), 7.26 (t, $^3J_{HH}$ = 7.8 Hz, 2H, $C_{Ar}H$), 7.14 (d, $^3J_{HH}$ = 7.8 Hz, 4H, $C_{Ar}H$), 7.02- 6.97 (m, 9H, $C_{Ar}H$), 6.45 (s, 2H, C_4 and C_5), 2.97 (septet, $^3J_{HH}$ = 7.0 Hz, 4H, CH), 1.59 (d, $^3J_{HH}$ = 7.0 Hz, 12H, CH_3), 1.97 (d, $^3J_{HH}$ = 7.0 Hz, 12H, CH_3). **$^{31}P\{^1H\}$ NMR (121 MHz, C_6D_6, 298K):** δ (ppm) = 30.6. This data is in accordance with that reported in the literature.[1]

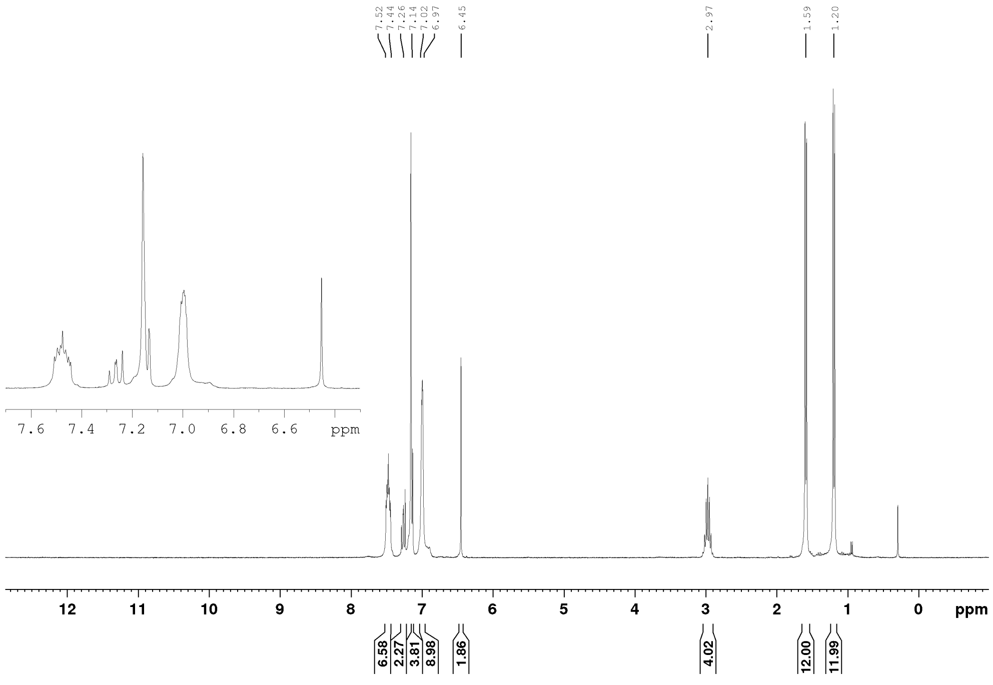

Figure **S1**. ^{1}H NMR spectrum of **[Pd(IPr)(PPh_3)]** in C_6D_6.

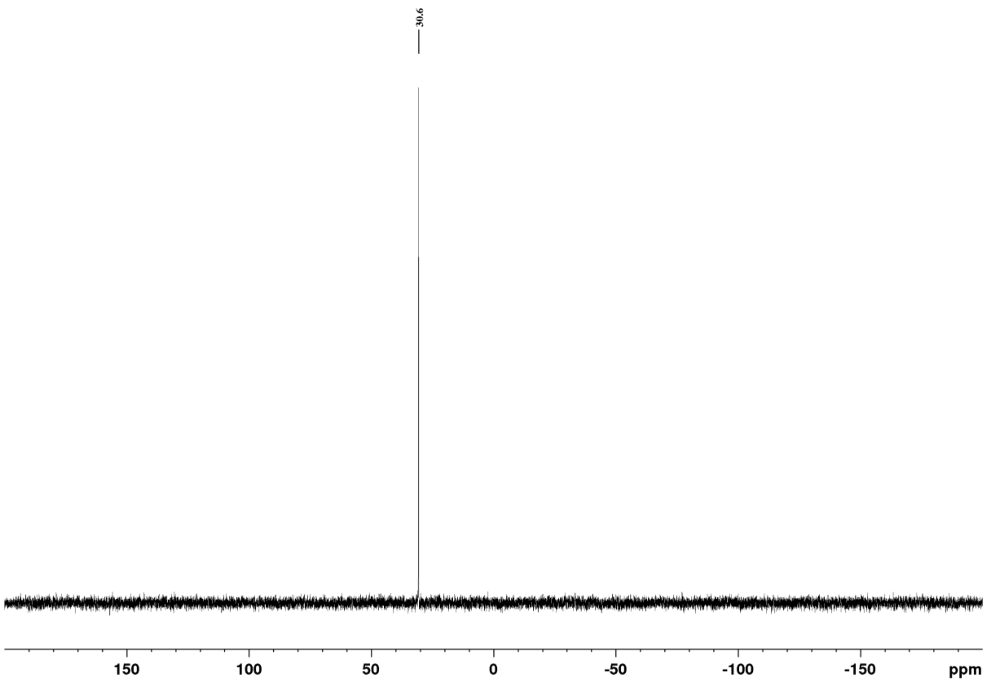

Figure **S2**. $^{31}P\{^1H\}$ NMR spectrum of **[Pd(IPr)(PPh$_3$)]** in C_6D_6.

V Free	V Buried	V Total	V Exact
96.2	83.3	179.5	179.6

%V_Free	%V_Bur	% Tot/Ex
53.6	46.4	100.0

xy	V_f	V_b	V_t	%V_f	%V_b
--	25.4	19.5	44.9	56.5	43.46
-+	24.8	20.0	44.9	55.4	44.65
++	18.0	26.9	44.9	40.1	59.88
+-	28.0	16.9	44.9	62.4	37.58

Steric Map

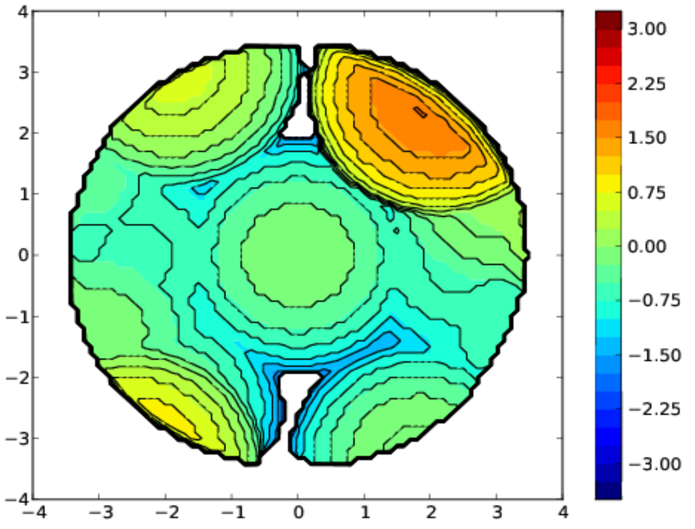

Figure **S3**. %V_{Bur} of **IPr** in **[Pd(IPr)(PPh$_3$)]**

V Free	V Buried	V Total	V Exact
124.5	55.1	179.5	179.6

%V_Free	%V_Bur	% Tot/Ex
69.3	30.7	100.0

xy	V_f	V_b	V_t	%V_f	%V_b
--	30.7	14.2	44.9	68.4	31.57
-+	30.6	14.3	44.9	68.1	31.89
++	31.8	13.1	44.9	70.8	29.21
+-	31.4	13.5	44.9	70.0	29.98

Steric Map

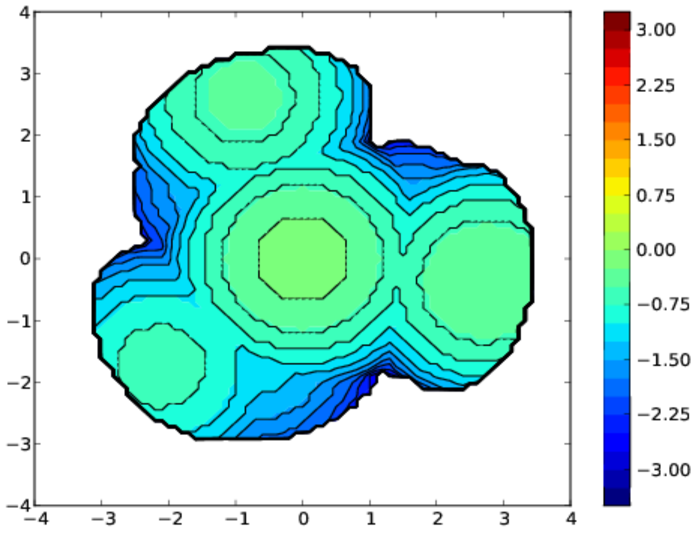

Figure **S4**. $\%V_{Bur}$ of $\mathbf{PPh_3}$ in $\mathbf{[Pd(IPr)(PPh_3)]}$

[Pd(IPr)(PCy$_3$)], 2: yellow powder. **Yield**: 85%. **^{1}H NMR (400 MHz, C_6D_6, 298K):** δ (ppm) = 7.28 (t, $^3J_{HH}$ = 7.8 Hz, 2H, C_{Ar}H), 7.18 (d, $^3J_{HH}$ = 7.8 Hz, 4H, C_{Ar}H), 6.47 (s, 2H, C_4 and C_5), 3.00 (septet, $^3J_{HH}$ = 7.0 Hz, 4H, CH), 1.86- 1.81 (m, 6H, CH), 1.77- 1.64 (m, 21H, CH), 1.50- 1.42 (m, 3H, CH), 1.31- 1.17 (m, 27H, CH). **^{31}P{^{1}H} NMR (202 MHz, C_6D_6, 298K):** δ (ppm) = 46.3

This data is in accordance with that reported in the literature.[1]

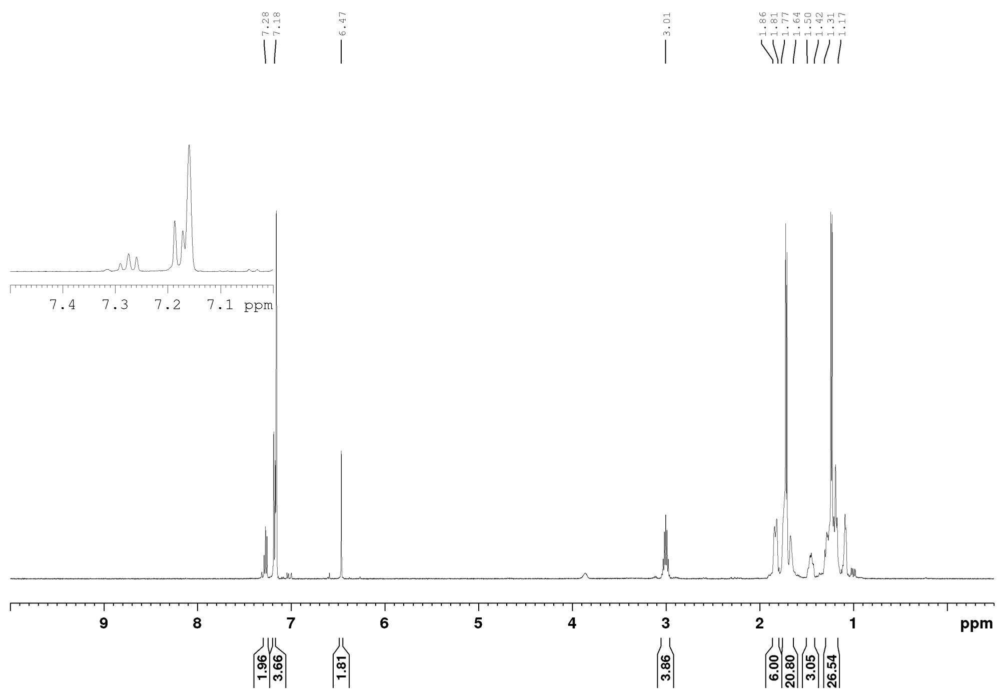

Figure **S5**. ^{1}H NMR spectrum of **[Pd(IPr)(PCy$_3$)]** in C_6D_6.

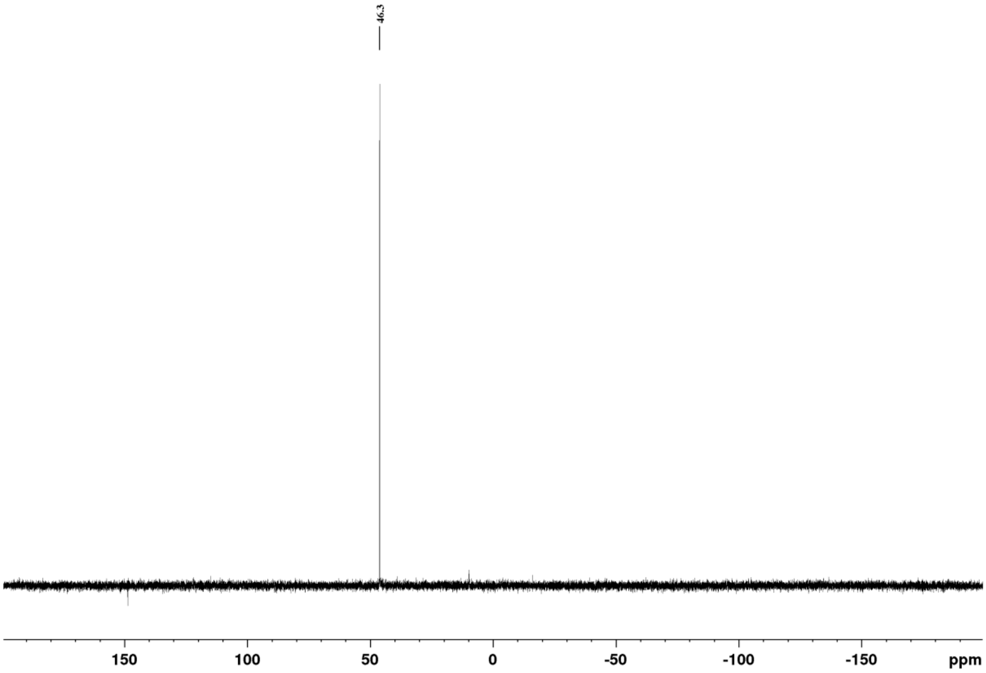

Figure **S6**. ^{31}P{^{1}**H**} NMR spectrum of **[Pd(IPr)(PCy$_3$)]** in C_6D_6.

V Free	V Buried	V Total	V Exact
93.4	86.1	179.5	179.6

%V_Free	**%V_Bur**	% Tot/Ex
52.0	**48.0**	100.0

xy	V_f	V_b	V_t	%V_f	**%V_b**
--	21.4	23.5	44.9	47.6	**52.38**
-+	24.7	20.2	44.9	55.0	**45.03**
++	25.7	19.2	44.9	57.3	**42.75**
+-	21.7	23.2	44.9	48.3	**51.71**

Steric Map

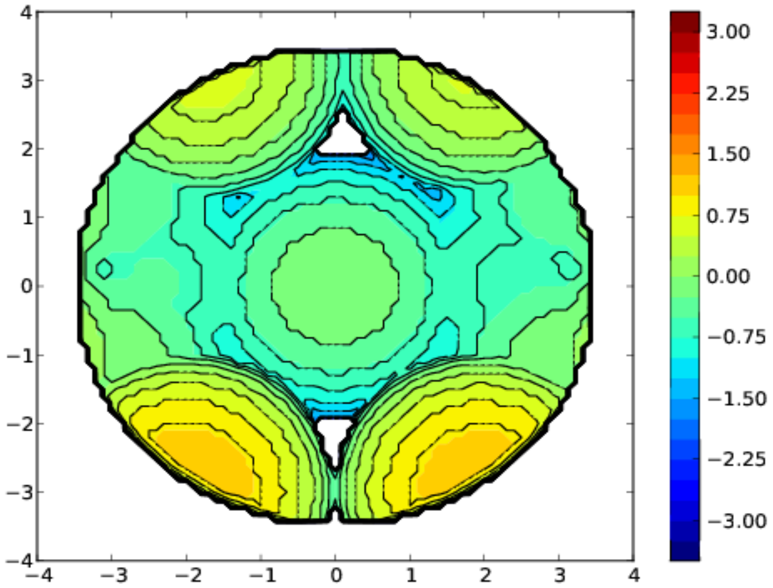

Figure **S7**. %V_{Bur} of **IPr** in **[Pd(IPr)(PCy$_3$)]**

V Free	V Buried	V Total	V Exact
119.7	59.8	179.5	179.6

%V_Free	**%V_Bur**	% Tot/Ex
66.7	**33.3**	100.0

xy	V_f	V_b	V_t	%V_f	**%V_b**
--	29.9	15.0	44.9	66.7	**33.32**
-+	28.2	16.7	44.9	62.9	**37.15**
++	33.0	11.9	44.9	73.6	**26.43**
+-	28.6	16.3	44.9	63.8	**36.23**

Steric Map

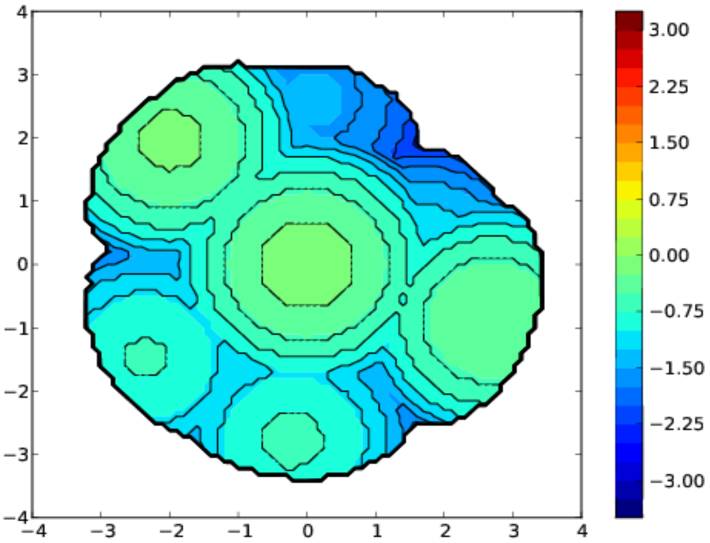

Figure **S8**. %V_{Bur} of **PCy$_3$** in **[Pd(IPr)(PCy$_3$)]**

[Pd(SIPr)(PCy$_3$)], 3: yellow powder. **Yield**: 83%. **^{1}H NMR (400 MHz, C$_6$D$_6$, 298K):** δ (ppm) = 7.25 (t, $^3J_{HH}$ = 7.7 Hz, 2H, C$_{Ar}$H), 7.17 (d, $^3J_{HH}$ = 7.7 Hz, 4H, C$_{Ar}$H), 3.37 (s, 4H, C$_4$ and C$_5$), 3.32 (septet, $^3J_{HH}$ = 6.9 Hz, 4H, CH), 1.8- 1.64 (m, 27H,CH), 1.46- 1.37 (m, 3H, CH), 1.34 (d, $^3J_{HH}$ = 6.9 Hz, 12H, CH$_3$), 1.24- 1.15 (m, 15H, CH). **^{31}P{^{1}H} NMR (202 MHz, C$_6$D$_6$, 298K):** δ (ppm) = 44.9. This data is in accordance with that reported in the literature.[1]

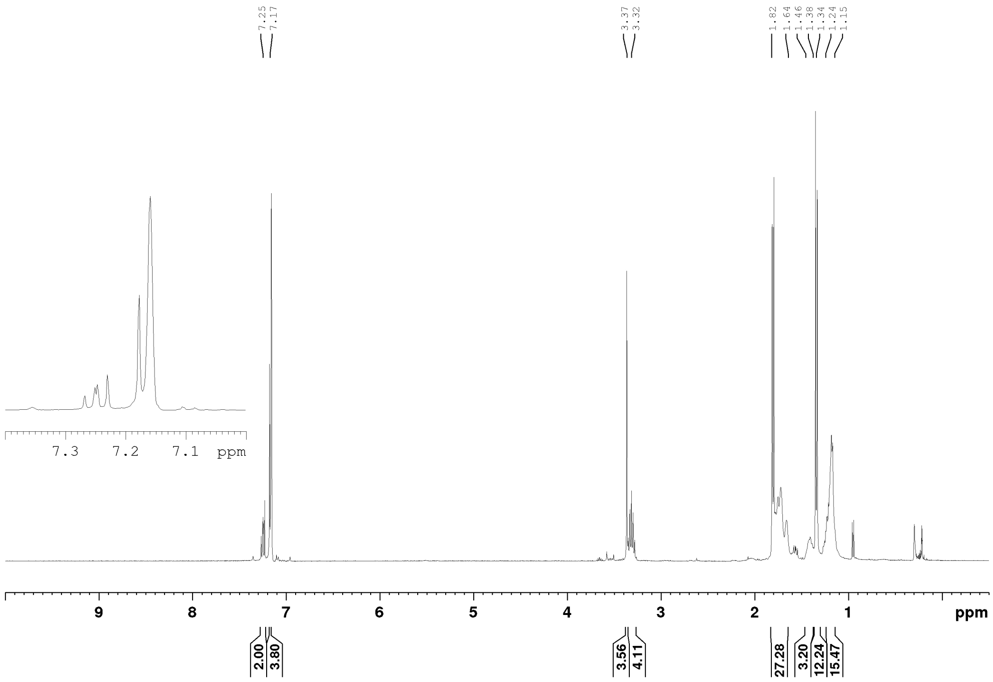

Figure **S9**. ^{1}H NMR spectrum of **[Pd(SIPr)(PCy$_3$)]** in C$_6$D$_6$.

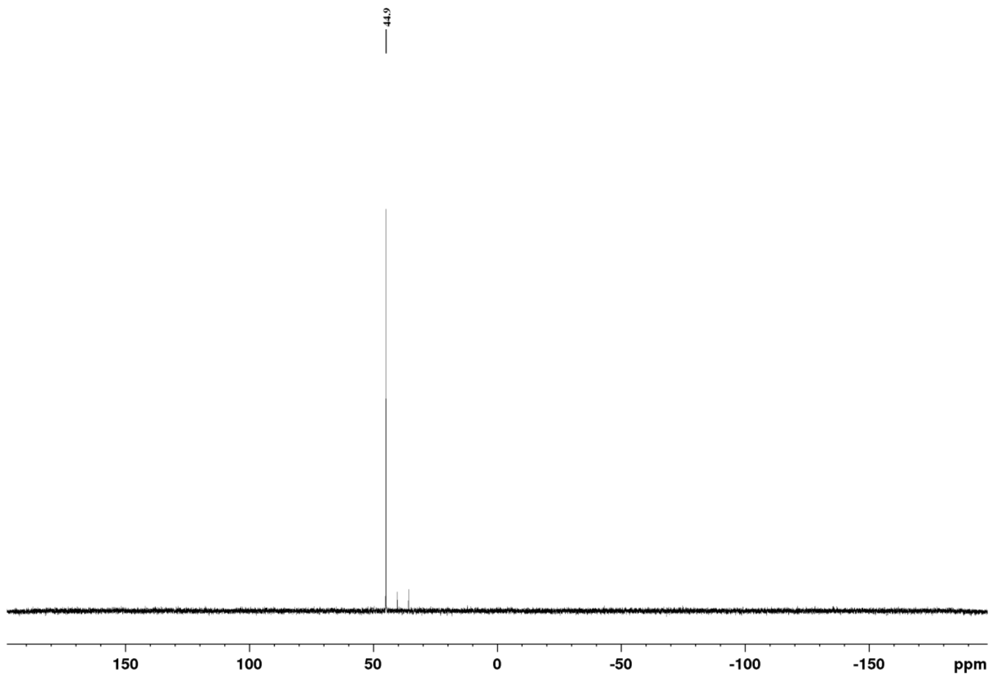

Figure **S10**. ^{31}P{^{1}**H**} NMR spectrum of **[Pd(SIPr)(PCy$_3$)]** in C_6D_6.

[Pd(IPr*)(PCy_3)], 4: yellow powder. **Yield**: 82%. **^{1}H NMR (300 MHz, C_6D_6, 298K):** δ (ppm)= 7.82 (d, $^3J_{HH}$ = 7.8 Hz, 8H, C_{Ar}H), 7.29 (t, $^3J_{HH}$ = 7.8 Hz, 8H, C_{Ar}H), 7.14- 7.03 (m, 9H, C_{Ar}H), 7.00- 6.90 (m, 19H, C_{Ar}H), 6.20 (s, 4H, $CHPh_2$), 5.27 (s, 2H, C_4 and C_5), 2.20- 2.10 (m, 6H, CH), 1.98 (s, 9H, CH and CH_3), 1.99- 1.70 (m, 7H, CH), 1.66- 1.52 (m, 7H, CH), 1.38- 1.23 (m, 7H, CH), 1.15- 0.99 (m, 4H, CH). **^{13}C{^{1}H} NMR (75 MHz, C_6D_6, 298K):** δ (ppm) = 199.9 (d, *J*= 90.9 Hz, C_2), 145.5 (s, C^{IV}), 144.3 (s, C^{IV}), 142.4 (s, C^{IV}), 138.0 (s, C^{IV}), 131.0 (s, CH), 130.0 (s, CH), 129.5 (s, CH), 128.6 (s, CH), 126.6 (s, CH), 126.6 (s, CH), 121.3 (s, C_4 and C_5), 51.8 (s, $CHPh_2$), 34.9 (d , *J*= 6.0 Hz, CH), 30.4 (s, CH), 28.3 (d, *J*= 10.5 Hz, CH), 27.7 (*J*= 11.2 Hz, CH), 27.0 (s, CH), 21.6 (s, CH_3). **^{31}P{^{1}H} NMR (121 MHz, C_6D_6, 298K):** δ (ppm) = 44.4. **Elemental analysis calcd for $C_{87}H_{89}N_2PPd$:** C, 80.38; H, 6.90; N, 2.15; found: C, 80.19; H, 6.85; N, 2.06.

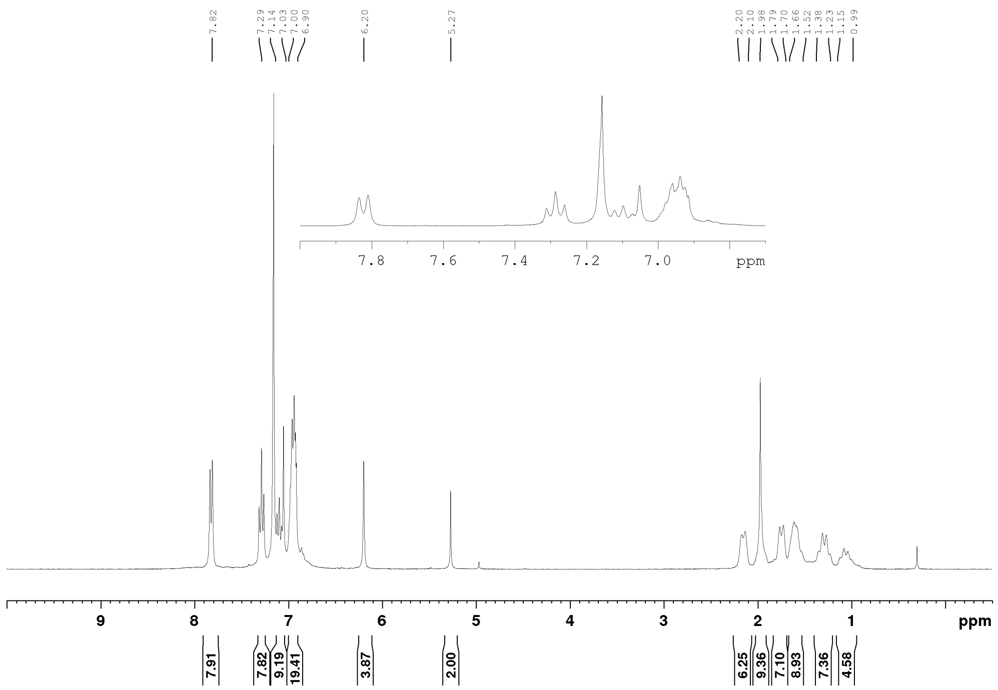

Figure **S11**. ^{1}H NMR spectrum of **[Pd(IPr*)(PCy_3)]** in C_6D_6.

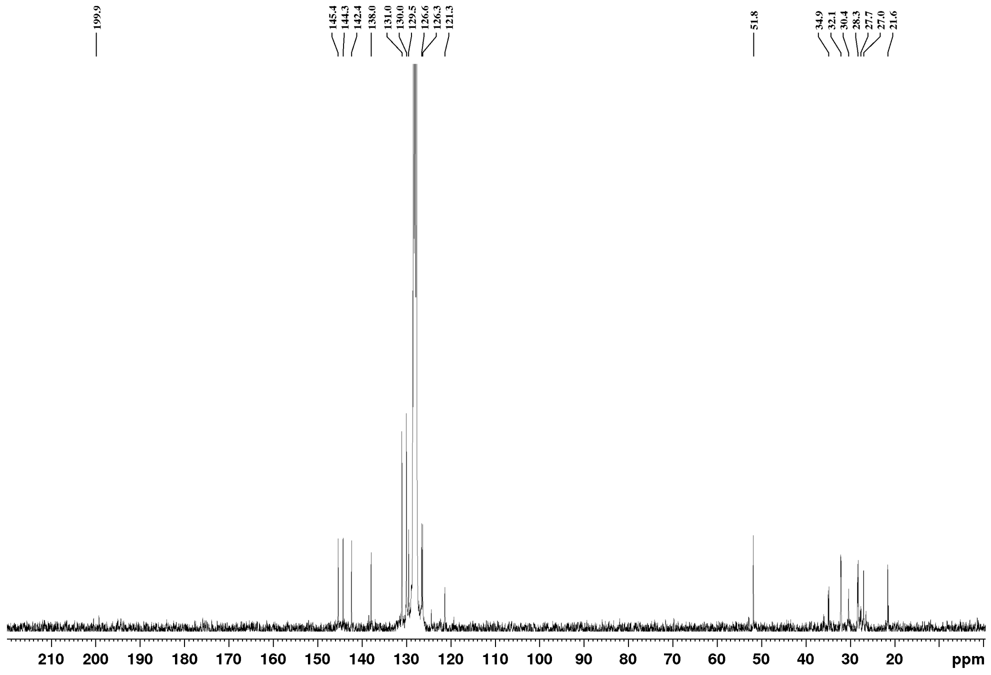

Figure **S12**. ^{13}C{^{1}**H**} NMR spectrum of **[Pd(IPr*)(PCy$_3$)]** in C_6D_6.

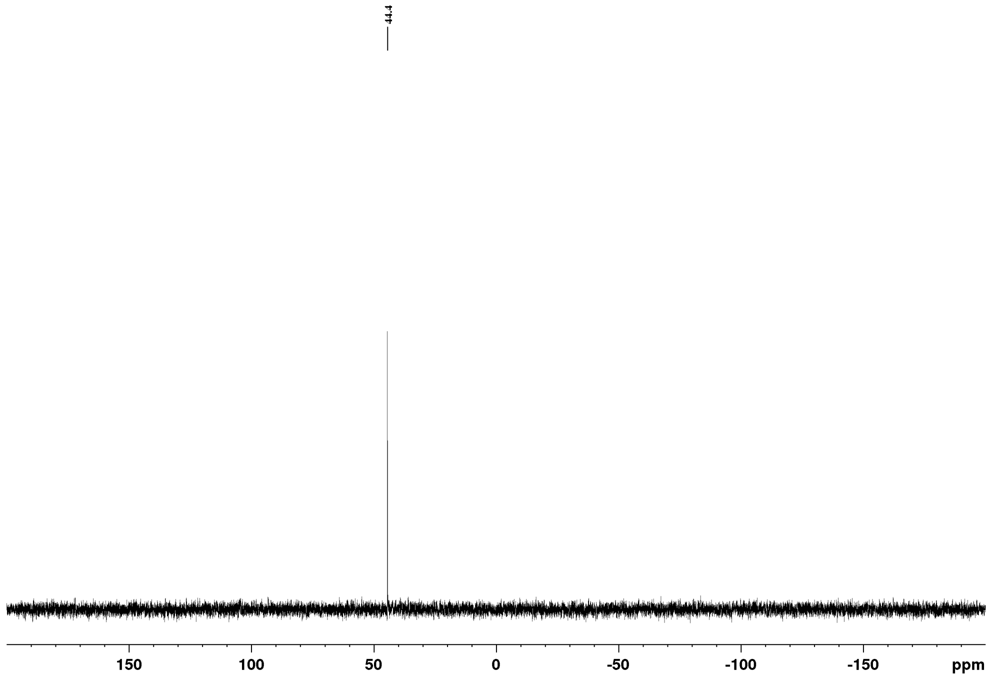

Figure **S13**. ^{31}P{^{1}**H**} NMR spectrum of **[Pd(IPr*)(PCy$_3$)]** in C_6D_6.

V Free	V Buried	V Total	V Exact
102.3	77.2	179.5	179.6

%V_Free	**%V_Bur**	% Tot/Ex
57.0	**43.0**	100.0

xy	V_f	V_b	V_t	%V_f	**%V_b**
--	29.0	15.9	44.9	64.7	**35.32**
-+	20.6	24.3	44.9	45.9	**54.08**
++	26.5	18.4	44.9	59.0	**41.02**
+-	26.2	18.7	44.9	58.3	**41.66**

Steric Map

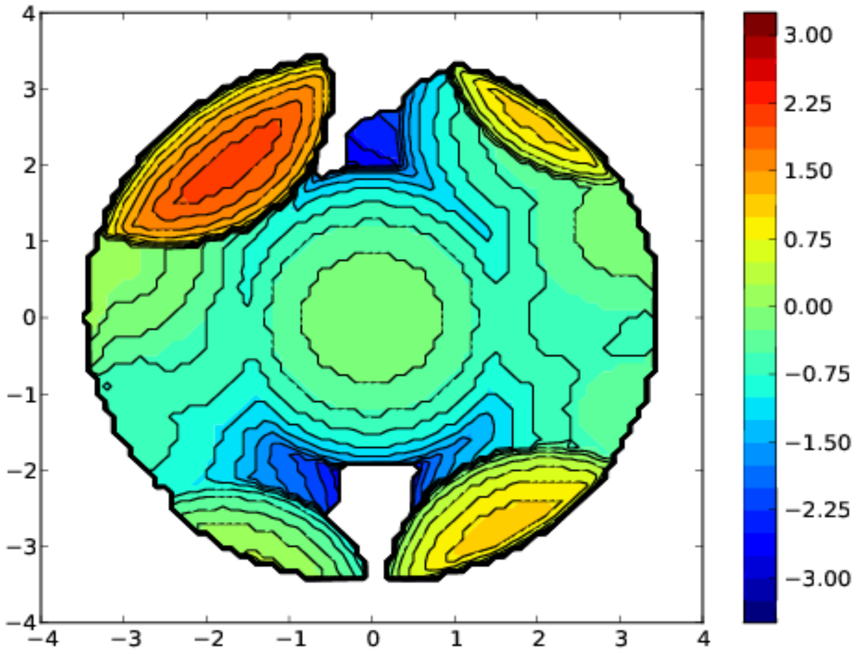

Figure **S14**. $\%V_{Bur}$ of **IPr*** in **[Pd(IPr*)(PCy$_3$)]**

V Free	V Buried	V Total	V Exact
120.6	58.9	179.5	179.6

%V_Free	**%V_Bur**	% Tot/Ex
67.2	**32.8**	100.0

xy	V_f	V_b	V_t	%V_f	**%V_b**
--	29.2	15.7	44.9	65.0	**34.98**
-+	29.5	15.4	44.9	65.7	**34.32**
++	29.2	15.7	44.9	65.0	**34.99**
+-	32.7	12.1	44.9	73.0	**27.01**

Steric Map

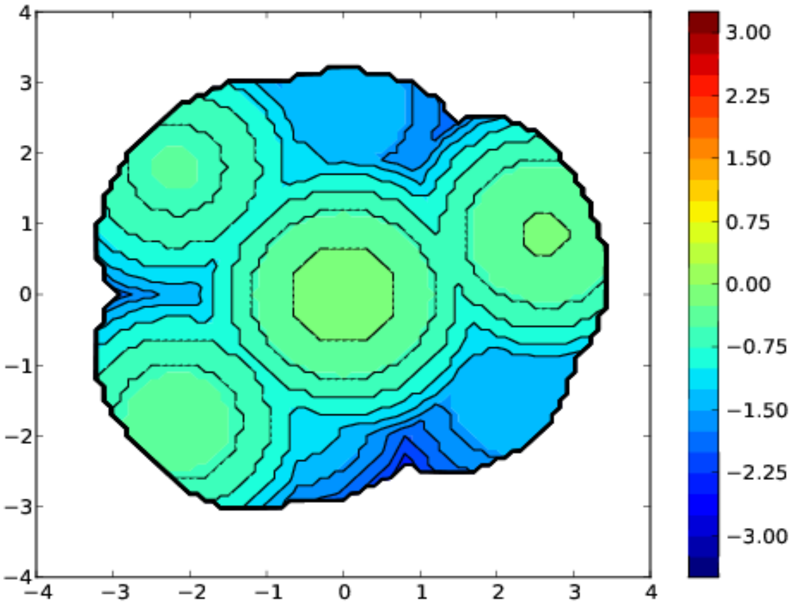

Figure **S15**. $\%V_{Bur}$ of **PCy$_3$** in **[Pd(IPr*)(PCy$_3$)]**

[Pd(IPr)$_2$], 5: orange powder. **Yield**: 81%. **^{1}H NMR (400 MHz, C_6D_6, 298K):** δ (ppm) = 7.30 (t, $^3J_{HH}$ = 7.7 Hz, 4H, $C_{Ar}H$), 7.10 (d, $^3J_{HH}$ = 7.7 Hz, 8H, $C_{Ar}H$), 6.27 (s, 4H, C_4 and C_5), 2.89 (septet, $^3J_{HH}$ = 6.8 Hz, 8H, CH), 1.22 (d, $^3J_{HH}$ = 6.8 Hz, 24H, CH_3), 1.12 (d, $^3J_{HH}$ = 6.8 Hz, 24H, CH_3). This data is in accordance with that reported in the literature.[1]

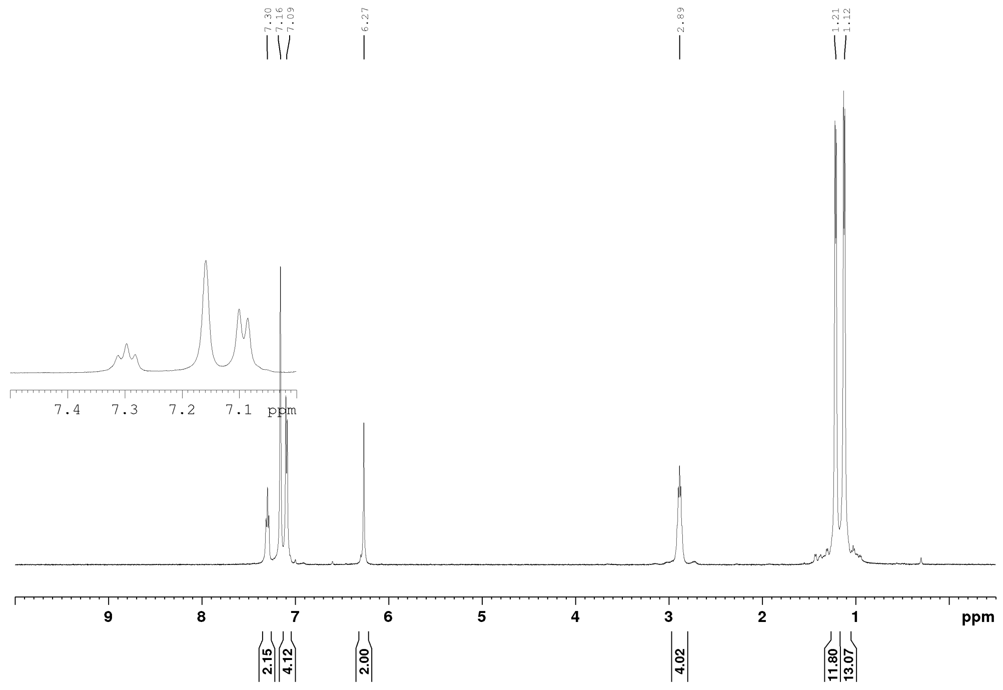

Figure **S16**. ^{1}H NMR spectrum of **[Pd(IPr)$_2$]** in C_6D_6.

V Free	V Buried	V Total	V Exact
103.0	76.6	179.5	179.6

%V_Free	**%V_Bur**	% Tot/Ex
57.4	**42.6**	100.0

xy	V_f	V_b	V_t	%V_f	**%V_b**
--	27.0	17.9	44.9	60.1	**39.93**
-+	21.5	23.4	44.9	47.9	**52.08**
++	26.4	18.5	44.9	58.8	**41.19**
+-	28.1	16.8	44.9	62.6	**37.38**

Steric Map

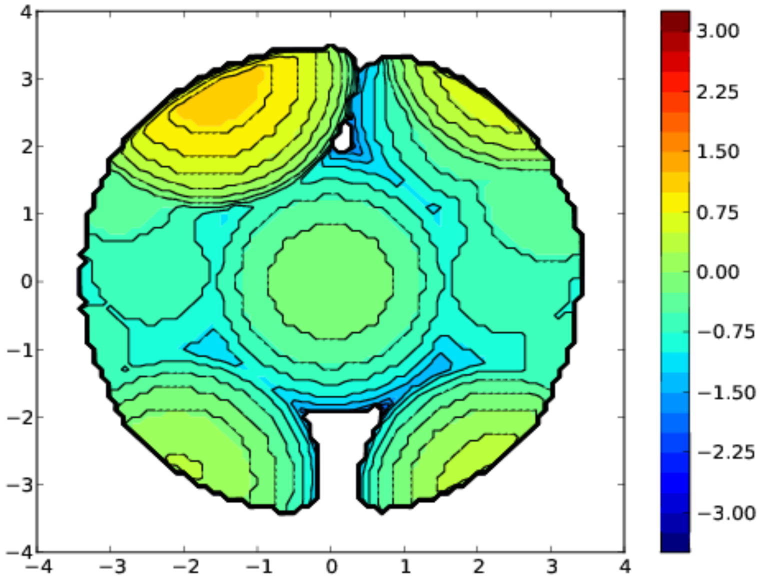

Figure **S17**. %V_{Bur} of **IPr** in **[Pd(IPr)$_2$]**

General procedure for the synthesis of complex [Pd(SIPr)$_2$], 6.[2] In a Schlenk flask, [Pd(μ-Cl)(η^3-crotyl)]$_2$ (0.508 mmol), sodium dimethylmalonate (1.02 mmol) and free SIPr (2.03 mmol) were dissolved in THF (40 mL). The reaction mixture was stirred during 16 h at 60°C. The solution was dried under vacuum and the solid was dissolved in toluene (15 mL) and filtered to remove insoluble materials. The supernatant solution was concentrated, cooled to -35°C. The product was collected by filtration and obtained as an orange solid (590 mg). **Yield**: 66%. **^{1}H NMR (400 MHz, C_6D_6, 298K):** δ (ppm) = 7.26 (t, $^3J_{HH}$ = 7.6 Hz, 4H, $C_{Ar}H$), 7.08 (d, $^3J_{HH}$ = 7.6 Hz, 8H, $C_{Ar}H$), 3.18-3.10 (m, 16H, C_4 and C_5 and CH), 1.22 (d, $^3J_{HH}$ = 6.9 Hz, 48H, CH_3). This data is in accordance with that reported in the literature.[2]

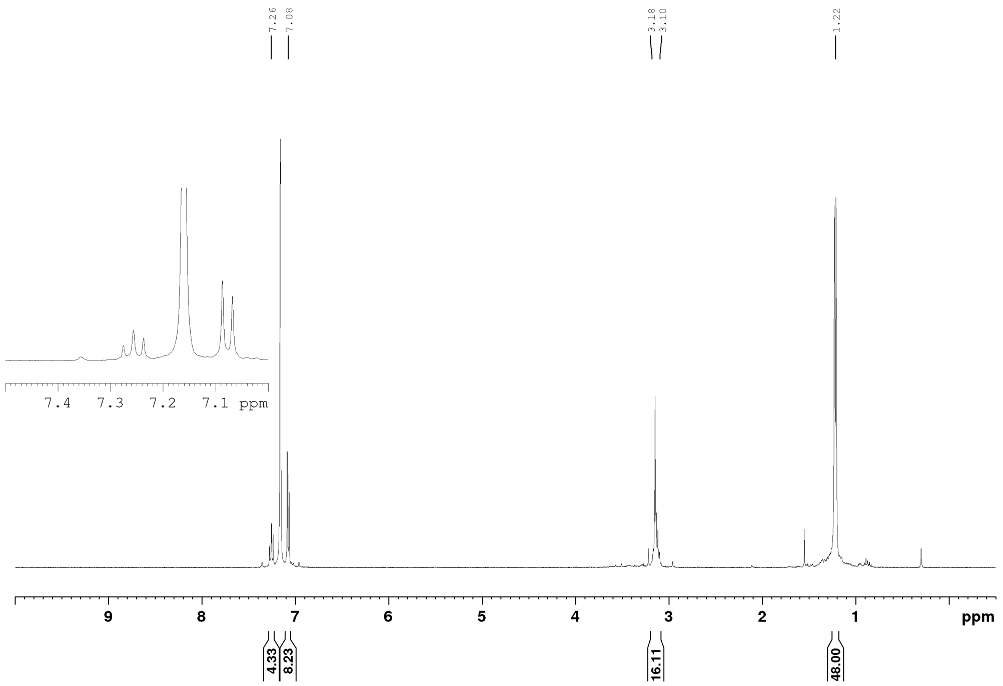

Figure **S18**. ^{1}H NMR spectrum of **[Pd(SIPr)$_2$]** in C_6D_6.

V Free	V Buried	V Total	V Exact
111.6	67.9	179.5	179.6

%V_Free	**%V_Bur**	% Tot/Ex
62.2	**37.8**	100.0

xy	V_f	V_b	V_t	%V_f	**%V_b**
--	32.2	12.7	44.9	71.7	**28.30**
-+	22.6	22.3	44.9	50.4	**49.65**
++	28.2	16.6	44.9	63.0	**37.05**
+-	28.6	16.3	44.9	63.7	**36.27**

Steric Map

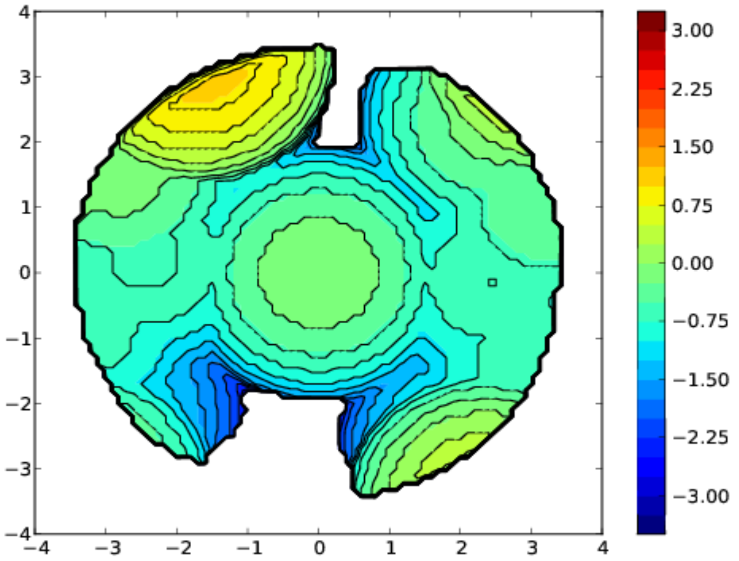

Figure **S19**. %V_{Bur} of **SIPr** in **[Pd(SIPr)$_2$]**

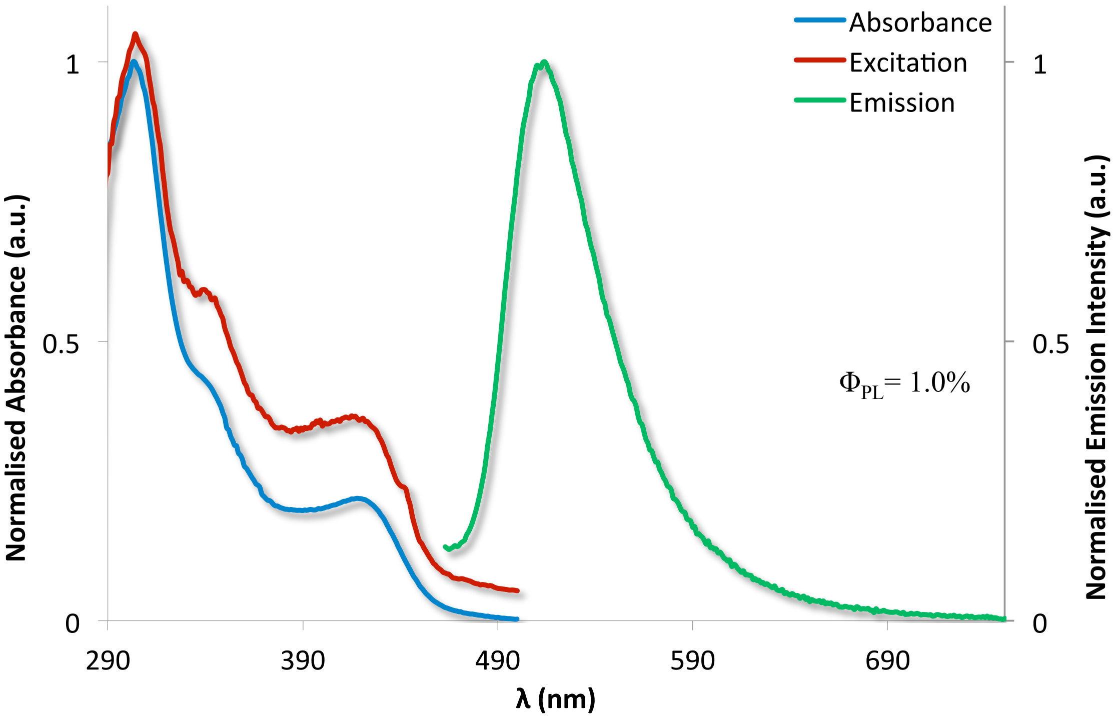

Figure **S20**. Summary of photophysical data for[Pd(IPr)(PPh$_3$)],**1**.. Normalized absorption, excitation and 298 K emission spectra in toluene, and Φ_{PL}.

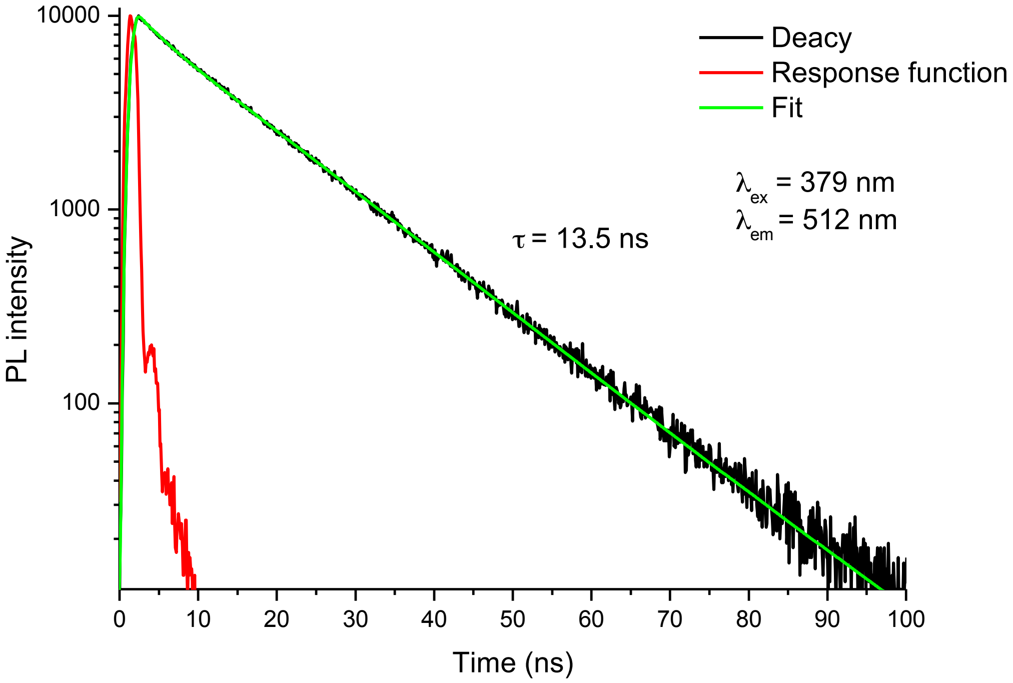

Figure **S21**. Lifetime spectra and fit for complex [Pd(IPr)(PPh_3)], **1**.

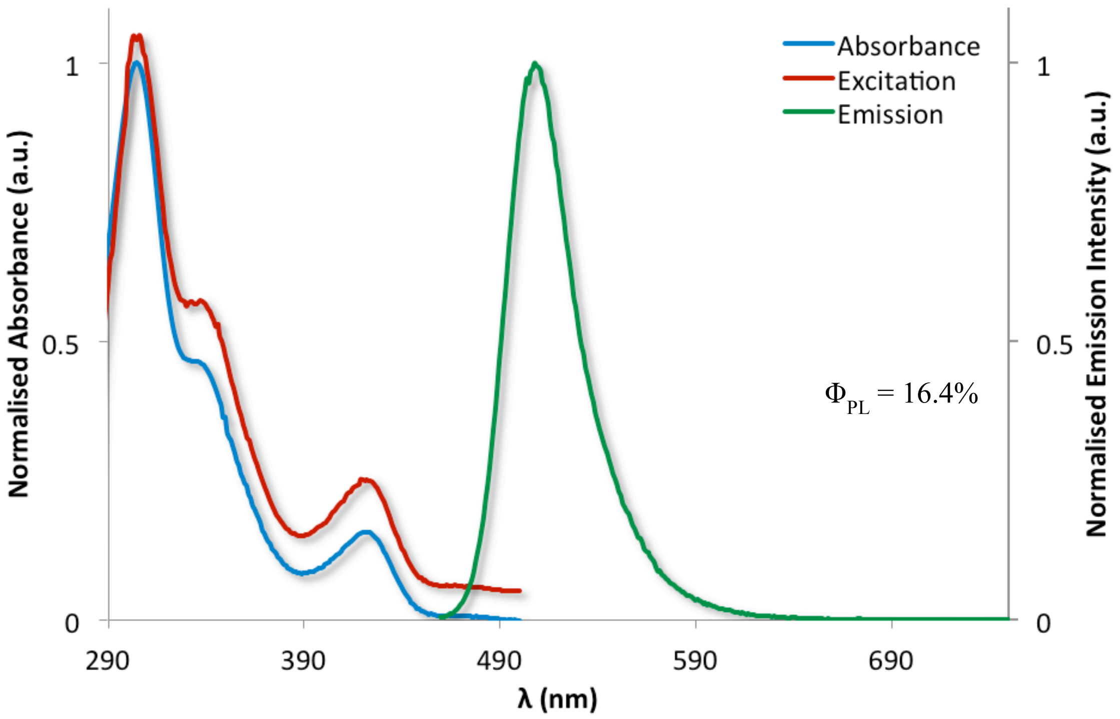

Figure **S22**. Summary of photophysical data for complex [Pd(IPr)(PCy_3)] **2**. Normalized absorption, excitation and 298 K emission spectra in toluene, and Φ_{PL}.

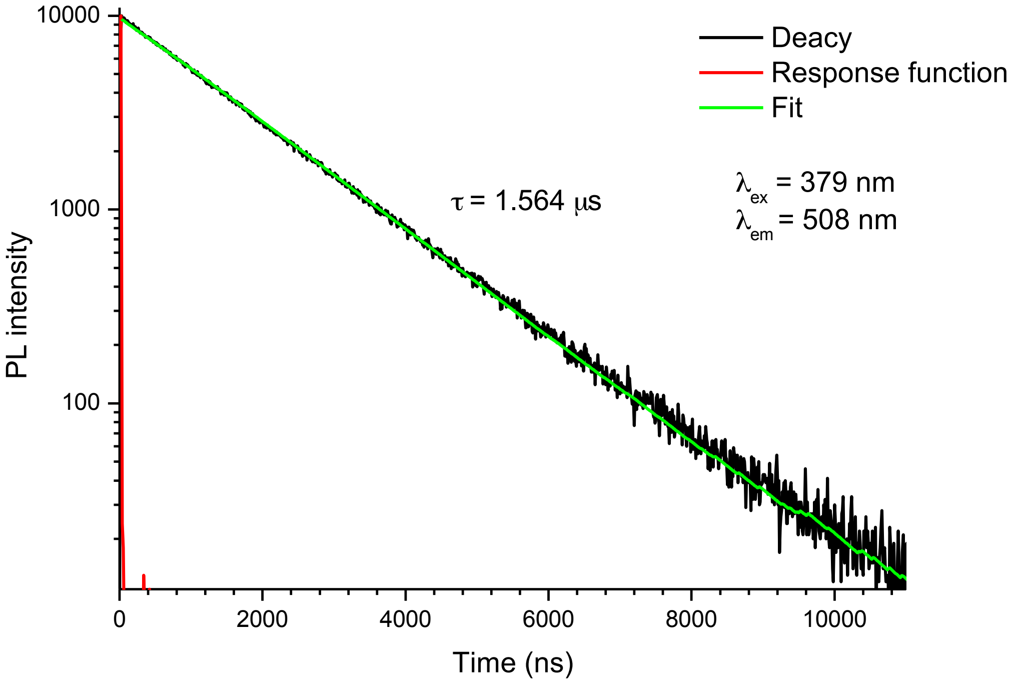

Figure **S23**. Lifetime spectra and fit for [Pd(IPr)(PCy_3)], **2**,.

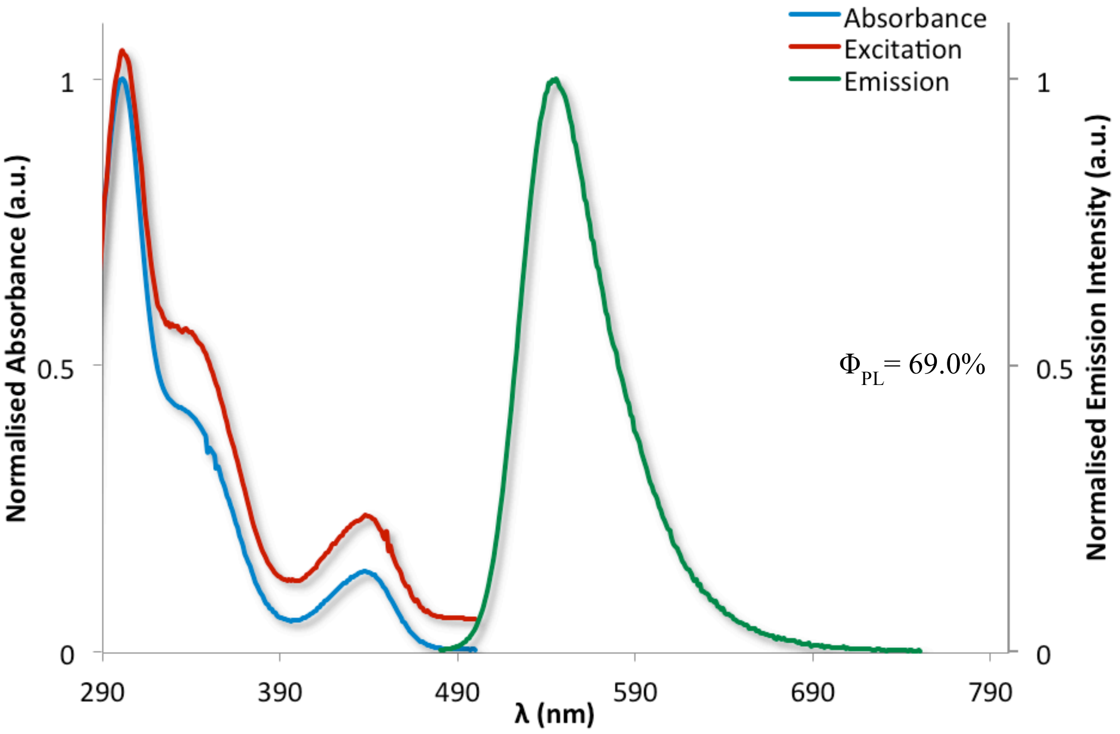

Figure **S24**. Summary of photophysical data for complex [Pd(SIPr)(PCy_3)], **3**. Normalized absorption, excitation and 298 K emission spectra in toluene, and Φ_{PL}.

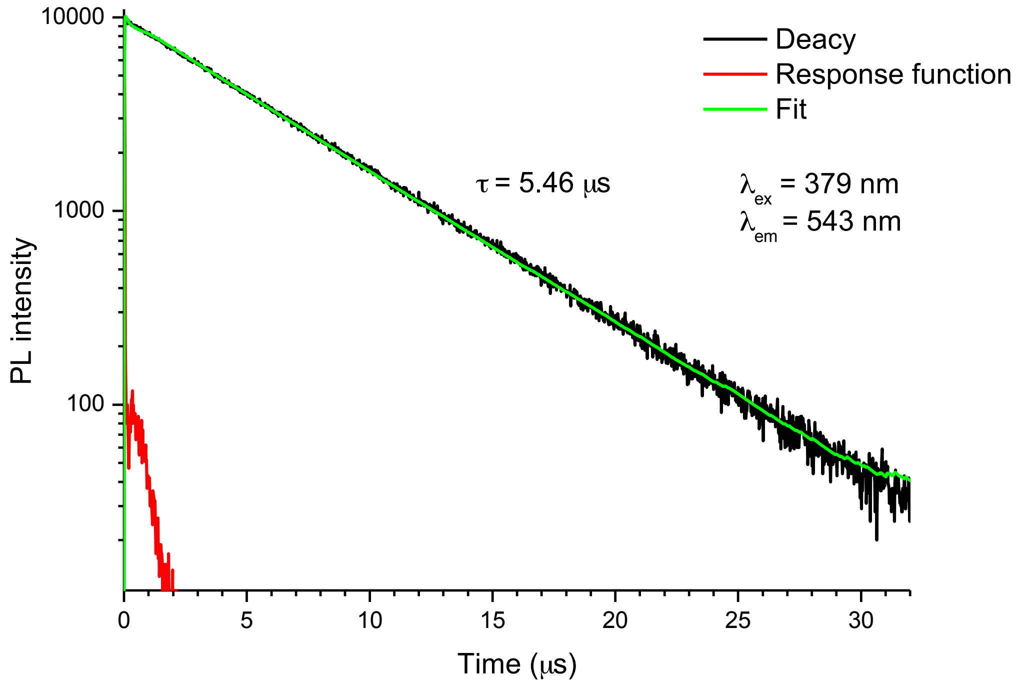

Figure **S25**. Lifetime spectra and fit for complex [Pd(SIPr)(PCy_3)], **3**.

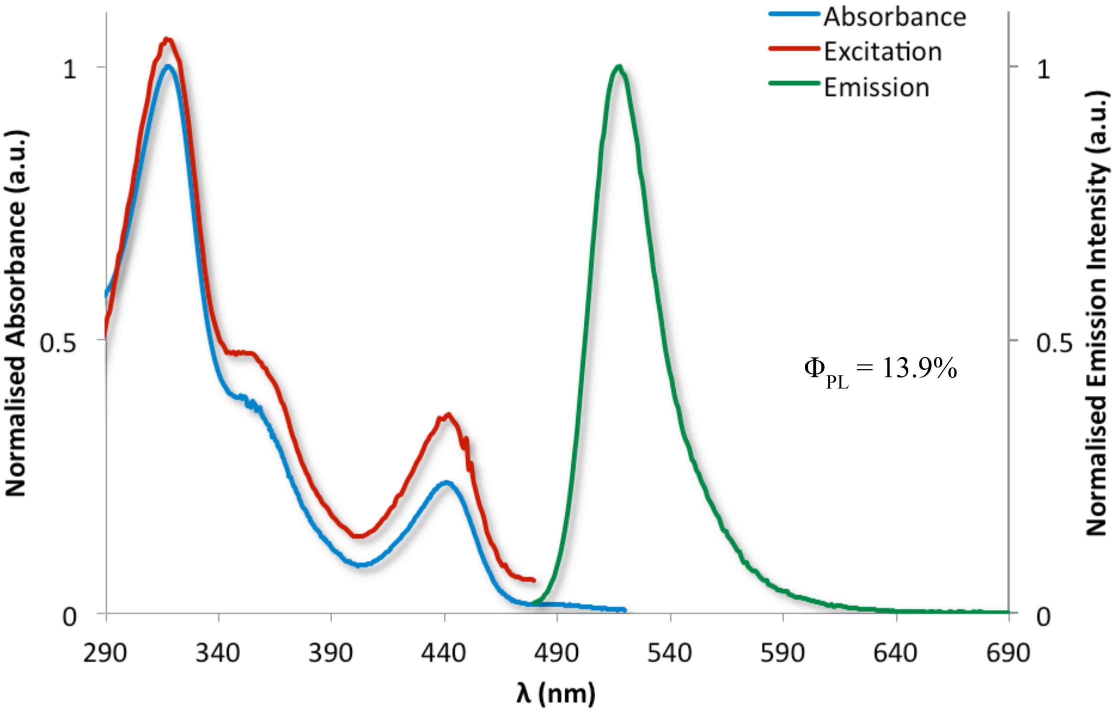

Figure **S26**. Summary of photophysical data for [Pd(IPr*)(PCy_3)], **4**. Normalized absorption, excitation and 298 K emission spectra in toluene, and Φ_{PL}.

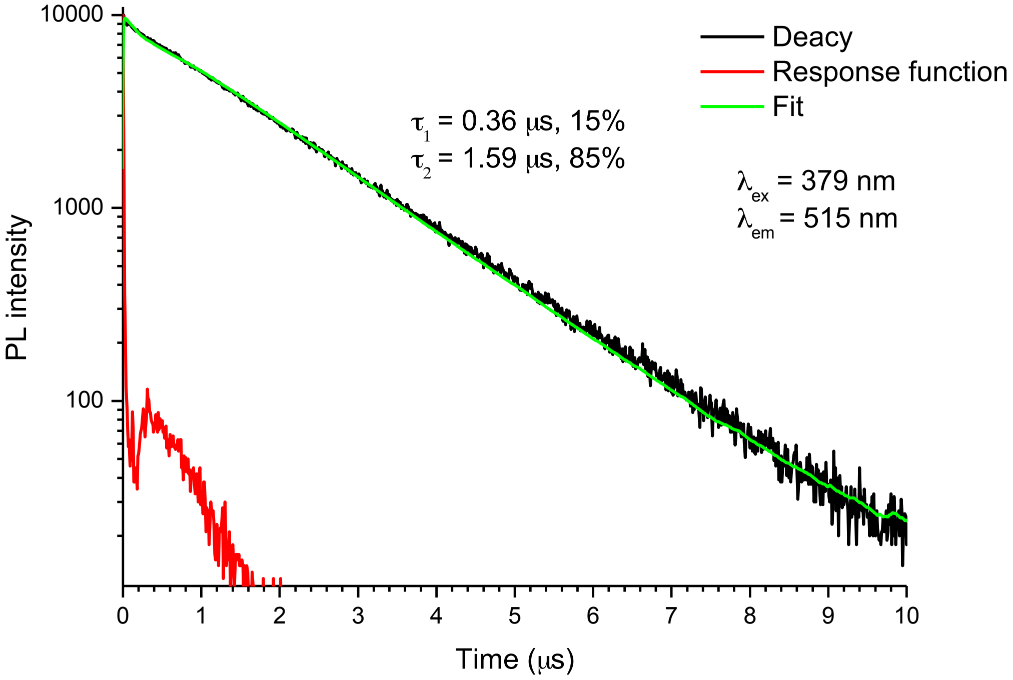

Figure **S27**. Lifetime spectra and fit for [Pd(IPr*)(PCy$_3$)], **4**.

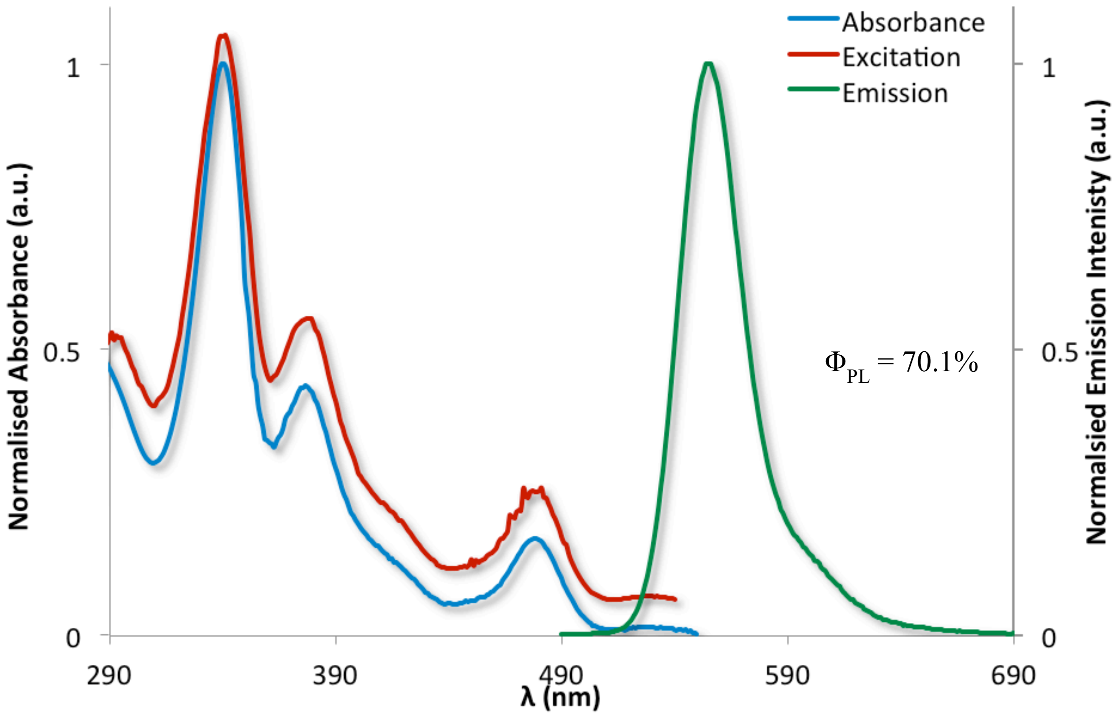

Figure **S28**. Summary of photophysical data for [Pd(IPr)$_2$] **5**. Normalized absorption, excitation and 298 K emission spectra in toluene, and Φ_{PL}.

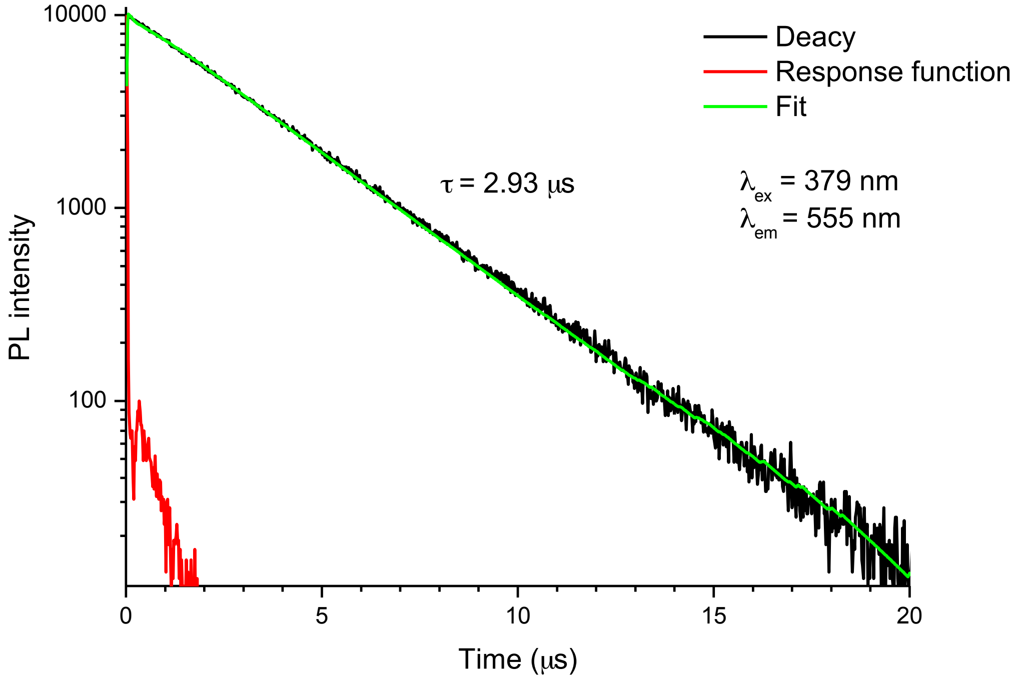

Figure **S29**. Lifetime spectra and fit for [Pd(IPr)$_2$] complex **5**.

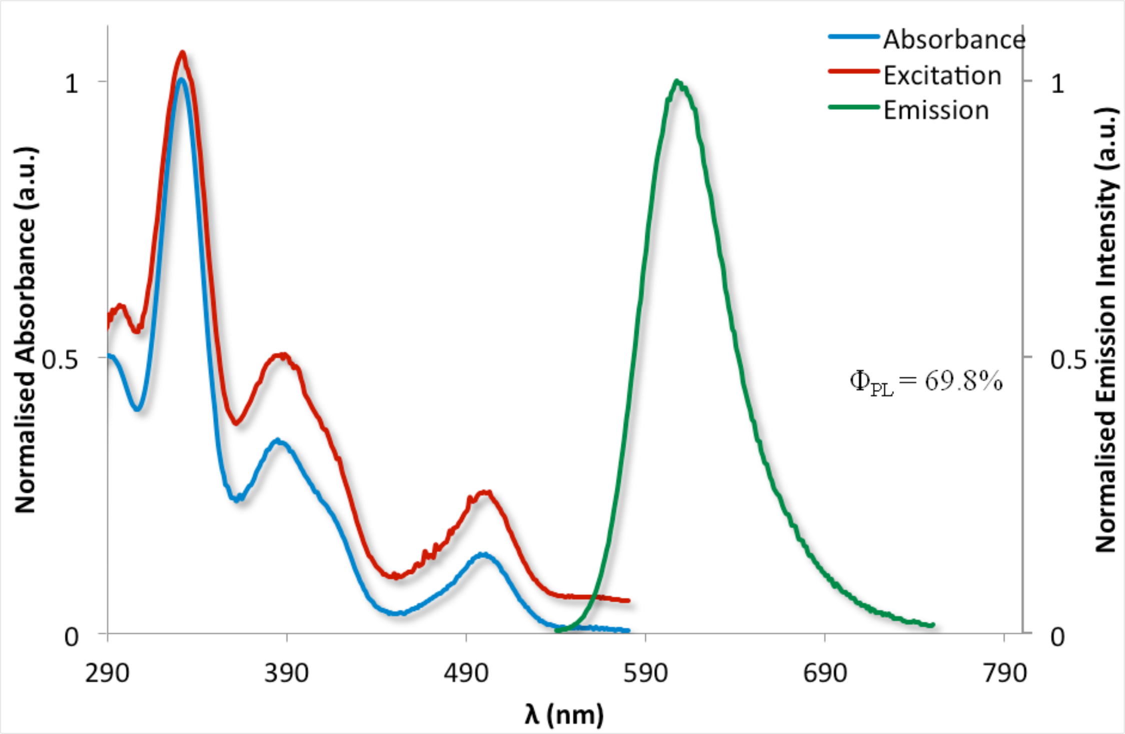

Figure **S30**. Summary of photophysical data for [Pd(SIPr)$_2$], **6**. Normalized absorption, excitation and 298 K emission spectra in toluene, and Φ_{PL}.

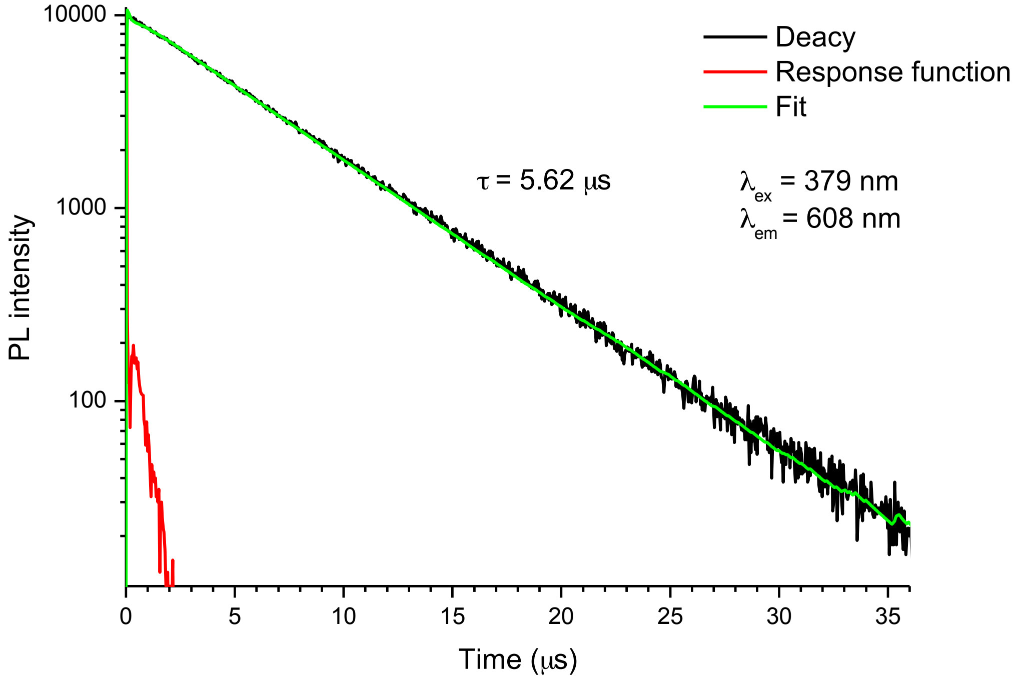

Figure **S31**. Lifetime spectra and fit for [Pd(SIPr)$_2$], **6**.

Table **S1**. Energy (in eV) and localisation of the frontier electronic levels ranging from HOMO-9 to LUMO+9. PPh3, PCy3, Ar, het, Pd refer to the triphenyl phosphine, the tricyclohexyl phosphine, the substituted phenyl rings on the periphery of the NHC ligands, the central rings of the NHC ligand and the palladium centre, respectively.

	1		2		3		4		5		6	
LUMO+9	0.15	PPh3	1.96	PCy3 (Ar/het)	1.92	PCy3 (Pd)	-0.08	Ar	0.99	het /Pd	0.94	het /Pd
LUMO+8	0.12	PPh3 (Ar/het)	1.52	het (Ar)	1.91	Pcy3 (Pd/het)	-0.10	Ar	0.36	het / Ar	0.36	het / Ar
LUMO+7	0.05	PPh3	1.39	het (Pd)	1.37	het (Pd)	-0.13	Ar	0.10	Ar	0.22	Ar
LUMO+6	-0.03	Ar	0.97	het	1.15	het	-0.16	Ar	0.08	Ar	0.17	Ar
LUMO+5	-0.11	Ar	0.92	PCy3	0.96	PCy3	-0.21	Ar	0.07	Ar	0.13	Ar
LUMO+4	-0.14	Ar (het)	0.20	Ar / het	0.22	Ar / het	-0.25	Ar	0.03	Ar	0.11	Ar
LUMO+3	-0.17	Ar	0.00	Ar	0.09	Ar	-0.35	Ar	-0.01	Ar	0.07	Ar
LUMO+2	-0.17	PPh3	-0.08	Ar	0.08	Ar	-0.37	Ar	-0.02	Ar	0.04	Ar
LUMO+1	-0.42	PPh3	-0.14	Ar	-0.06	Ar	-0.49	Ar	-0.04	Ar	0.03	Ar
LUMO	-0.50	PPh3	-0.16	Ar / het	-0.15	Ar / het	-0.50	Ar (het)	-0.14	het / Ar	-0.15	het / Ar
HOMO	-3.95	Pd	-3.76	Pd	-3.75	Pd	-3.85	Pd	-3.38	Pd	-3.42	Pd
HOMO-1	-4.58	Pd	-4.36	Pd	-4.39	Pd	-4.45	Pd	-4.02	Pd	-4.09	Pd
HOMO-2	-4.58	Pd	-4.37	Pd	-4.40	Pd	-4.45	Pd	-4.03	Pd	-4.10	Pd
HOMO-3	-5.07	Pd (het)	-4.83	Pd (het)	-4.92	Pd	-4.88	Pd (het)	-4.40	Pd (het)	-4.60	Pd
HOMO-4	-5.20	Pd	-4.91	Pd	-5.01	Pd (het)	-4.97	Pd	-4.53	Pd	-4.69	Pd (het)
HOMO-5	-6.16	PPh3 (Pd)	-6.29	PCy3 / het (Pd)	-6.06	het	-6.13	Ar	-6.27	Ar	-5.91	het
HOMO-6	-6.40	Ar	-6.37	Ar	-6.25	PCy3 / het (Pd)	-6.15	Ar	-6.28	Ar	-5.92	het
HOMO-7	-6.42	Ar	-6.39	Ar	-6.30	Ar	-6.23	Ar	-6.28	Ar	-6.19	Ar
HOMO-8	-6.62	PPh3	-6.63	Ar / het	-6.31	Ar	-6.28	Ar (het/PCy3)	-6.29	Ar	-6.20	Ar
HOMO-9	-6.62	PPh3	-6.70	het (Ar / Pd)	-6.50	Ar (het)	-6.36	PCy3 / het (Pd)	-6.43	het	-6.21	Ar

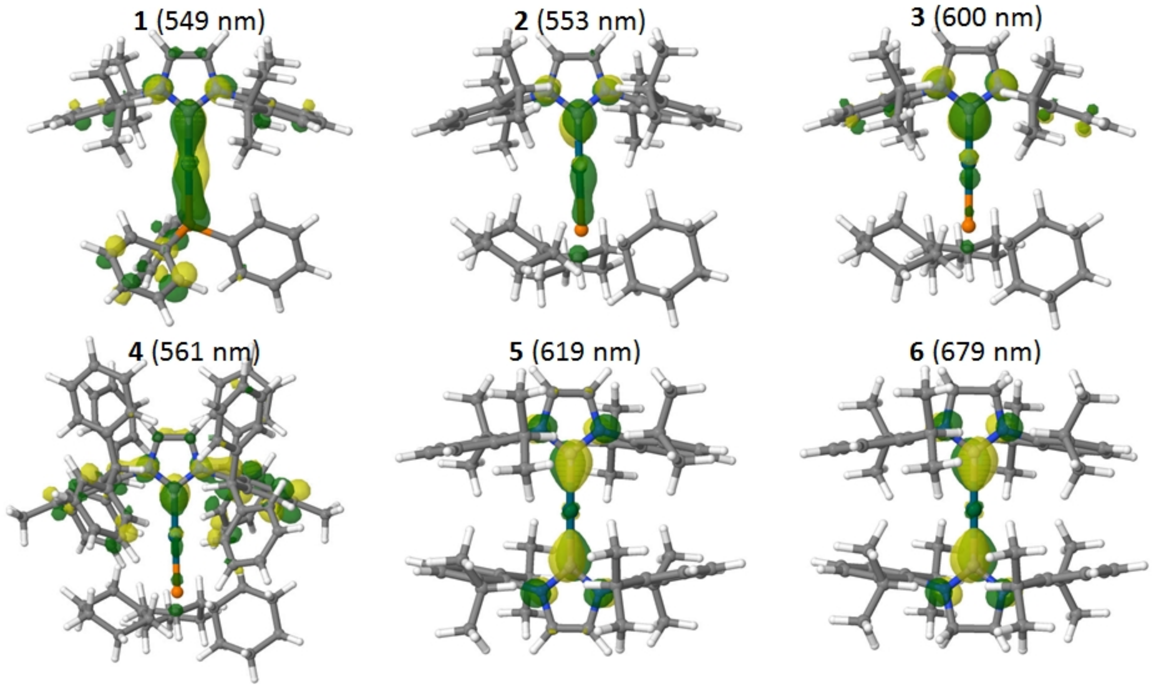

Figure **S32**. Description of the ligands involved in the emission process for complexes **1-6**. The yellow/green isosurfaces are generated by the Jmol program combining for each atom the LCAO coefficients in all the unoccupied molecular orbitals involved in the TD-DFT description of the lowest triplet excited state and their CI contributions. The spin densities on the Pd center are 1.13, 1.30, 1.20, 1.20, 1.24 and 1.20 for **1** to **6**, respectively.

References

(1). S. Fantasia and S. P. Nolan, *Chem. Eur. J.*, 2008, **14**, 6987-6993.

(2). L. R. Titcomb, S. Caddick, F. G. N. Cloke, D. J. Wilson and D. McKerrecher, *Chem. Commun.*, 2001, 1388-1389.
